# Photosensitization of TiO_2_ microspheres by novel Quinazoline-derivative as visible-light-harvesting antenna for enhanced Rhodamine B photodegradation

**DOI:** 10.1038/s41598-023-38497-9

**Published:** 2023-08-09

**Authors:** Mahmoud Adel Hamza, Sameh A. Rizk, Ezz-Elregal M. Ezz-Elregal, Shaimaa A. Abd El-Rahman, Sayed K. Ramadan, Zeinab M. Abou‑Gamra

**Affiliations:** Chemistry Department, Faculty of Science, Ain-Shams University, Abbassia, Cairo, Egypt

**Keywords:** Photocatalysis, Synthetic chemistry methodology, Pollution remediation

## Abstract

Water pollution is one of the global threats severely affecting our planet and human health. Organic textile dyes are one of the common organic water pollutants that are presentient to degradation by traditional physical methods. Semiconductor-assisted photocatalysis is considered a green, efficient, and sustainable technology for wastewater treatment. To maximize the efficient utilization of solar radiation, it is of pivotal significance to explore novel organic molecules to be employed as efficient dye sensitizers for wide-bandgap semiconductors to extend their performance to the Visible-light region. Hence, in this work, we are proposing the design and synthesis of novel structures of QAD molecule as a dye photosensitizer with extended visible light absorptivity due to the extended π–π/n–π conjugations, to promote the performance of TiO_2_ nanoparticles to the visible-light region and enhance the charge separation. The physicochemical characterizations confirmed the successful synthesis of QAD, TiO_2,_ and QAD/TiO_2_ samples with the proposed structures. The anchoring of QAD molecules on the surface of TiO_2_ caused a substantial improvement in the optical characteristics of TiO_2_ as well as overcoming its common drawbacks by decreasing its bandgap energy to 2.6 eV, a remarkable reduction of PL intensity indicating reducing the e–h recombination and enhancing the charge separation, and creation of efficient visible light-harvesting antenna in the range of 400–600 nm. Besides, the QAD/TiO_2_ sample achieved a 3-fold enhancement in the observed rate constant of the photodegradation of Rhodamine B dye compared to the bare TiO_2_. The parameters affecting the photodegradation process were optimized and the sample displayed outstanding stability after 4 consecutive cycles. Finally, the effect of the scavengers was investigated and ${\mathrm{O}}_{2}^{\cdot -}$ was proposed to be the most reactive species and the mechanism of the enhancement was suggested based on the electron injection from the QAD’s HOMO level to the TiO_2_’s CB. Finally, this work opens the door for various studies for the investigation of the proposed structures or similar structures in various photocatalytic/biomedical applications.

## Introduction

Visible-light-driven semiconductor-based photocatalysis has become one of the most successful green strategies for the effective harvesting and utilization of incident solar irradiation to catalyze the chemical process by converting the eternally accessible solar energy to valuable chemical energy to be applied in crucial purposes such as wastewater treatment, hydrogen production, ammonia production, solar cells, and carbon dioxide reduction^1–6^. Amongst various photocatalysts, TiO_2_ nanoparticles can be considered the most frequently employed semiconductors because of their availability, low cost, non-toxicity, outstanding photocatalytic activity, and long durability^7–12^. Nevertheless, the relatively wide bandgap energy (3.20 eV for Anatase) and fast e–h recombination rate hinder the industrial applications of bare TiO_2_ photocatalysts; this focused the light on the crucial need for finding affordable, robust, effective, and innovative visible-light-active photocatalysts^5,13,14^. In the last two decades, there were many attempts to enhance the photocatalytic behaviour of wide-bandgap photocatalysts (e.g. TiO_2_ and ZnO) by extending their optical absorbance to the visible-light range and improving the charge separation by different approaches including metal/nonmetal doping/co-doping^4,7^, semiconductor coupling^15–17^, coupling with carbon materials^8,18^, and dye Sensitization^19–21^.

As a simulation of the photosynthesis process in plants, dye sensitization is found to be one of the most promising approaches to improving the optical and photocatalytic characteristics of TiO_2_ nanoparticles^19–26^. In our previous works on porphyrin-based TiO_2_ nanoparticles^19,20^, we have noticed that the anchoring of tetra(4-carboxyphenyl)porphyrin (TCPP) caused a substantial enhancement in the whole optical characteristics and the photocatalytic performance of TiO_2_ photocatalyst by (1) decreasing the e–h recombination rates, (2) enhancement the lifetime, and (3) reducing the bandgap energy to about 2.6 eV, in addition to (4) establishing an outstanding visible-light-harvesting antenna to overcome their inactivity in the visible light range along with (5) doubling the photocatalytic activity of TCPP/TiO_2_ photocatalyst toward Rhodamine B (RB) photodegradation. Apart from the outstanding wide absorption capability of TCPP as a photosensitizer, it suffers from instability in alkaline media (pH > 10)^19^. Similarly, M. Sedghi^22^ et al. investigated the effect of TCPP to enhance the TiO_2_/Al and shift its response to the visible region; however, the achieved RB removal % was only 29.19%. E. Valadez-Renteria et al.^24^ employed green chlorophyll as an efficient photosensitizer to TiO_2_:W composite that efficiently degraded RB dye, but ~20% reduction in the activity was observed after the 3rd cycle. Zyoud et al.^23^ synthesized Anthocyanin-sensitized TiO_2_ nanoparticles for the efficient photodegradation of phenazopyridine under solar simulated light that achieve high degradation% (> 90%); however, the degradation% was declined to about 55% after the second use because of the loss of the photosensitizer.

Hence, the design and synthesis of novel efficient and stable organic dyes are one of the ideal solutions for designing efficient organic dye sensitizers with tunable properties to be employed in visible-light-driven semiconductor nanoparticles for various photocatalytic applications. Quinazoline-derivatives are a valuable class of fused heterocyclic compounds that are commonly employed in various fields such as pharmaceuticals and biomedicine for Alzheimer's disease^27^, Diabetes mellitus^28^, an inhibitor of COVID-19^29^, and SARS-CoV‑2^30^, and as potential bioactive Scaffold in medicinal chemistry^31^, in addition to the other general uses in material science and organic synthesis in general^32,33^. However, this class has not been employed as organic photosensitizers.

Thus, this work is reporting a facile synthesis of a novel organic compound (Quinazoline-derivative, QAD) to be used as a novel organic dye sensitizer for TiO_2_ nanoparticles. Besides, the synthesis of TiO_2_ using a simple template-free sol–gel approach and QAD-photosensitized TiO_2_ via a facile wet-impregnation route was explored and the corresponding physicochemical characterizations have been investigated. The photocatalytic activities of the as-fabricated samples towards the photodegradation of Rhodamine B (RB) dye were investigated and the parameters affecting the photodegradation were optimized. Finally, the mechanism of the boosted photocatalytic activity of QAD/TiO_2_ nanoparticles compared to the bare TiO_2_ nanoparticles was elucidated to clarify the outstanding role of incorporation of the proposed QAD molecules.

## Experimental methods

### Materials

Titanium tetra-(isopropoxide), methyl viologen (MV), p-benzoquinone (p-BQ), and iso-propyl alcohol (i-PrOH) were purchased from Sigma Aldrich. Rhodamine B dye (RB) was obtained from Merck, while methyl iodide was obtained from Prolabo. All other chemicals were pure and employed without the aid of any additional purifying processes. Distilled water was utilized for preparing the aqueous solutions of RB.

### Synthesis of novel Quinazoline-derivative photosensitizer

**Figure 1:**
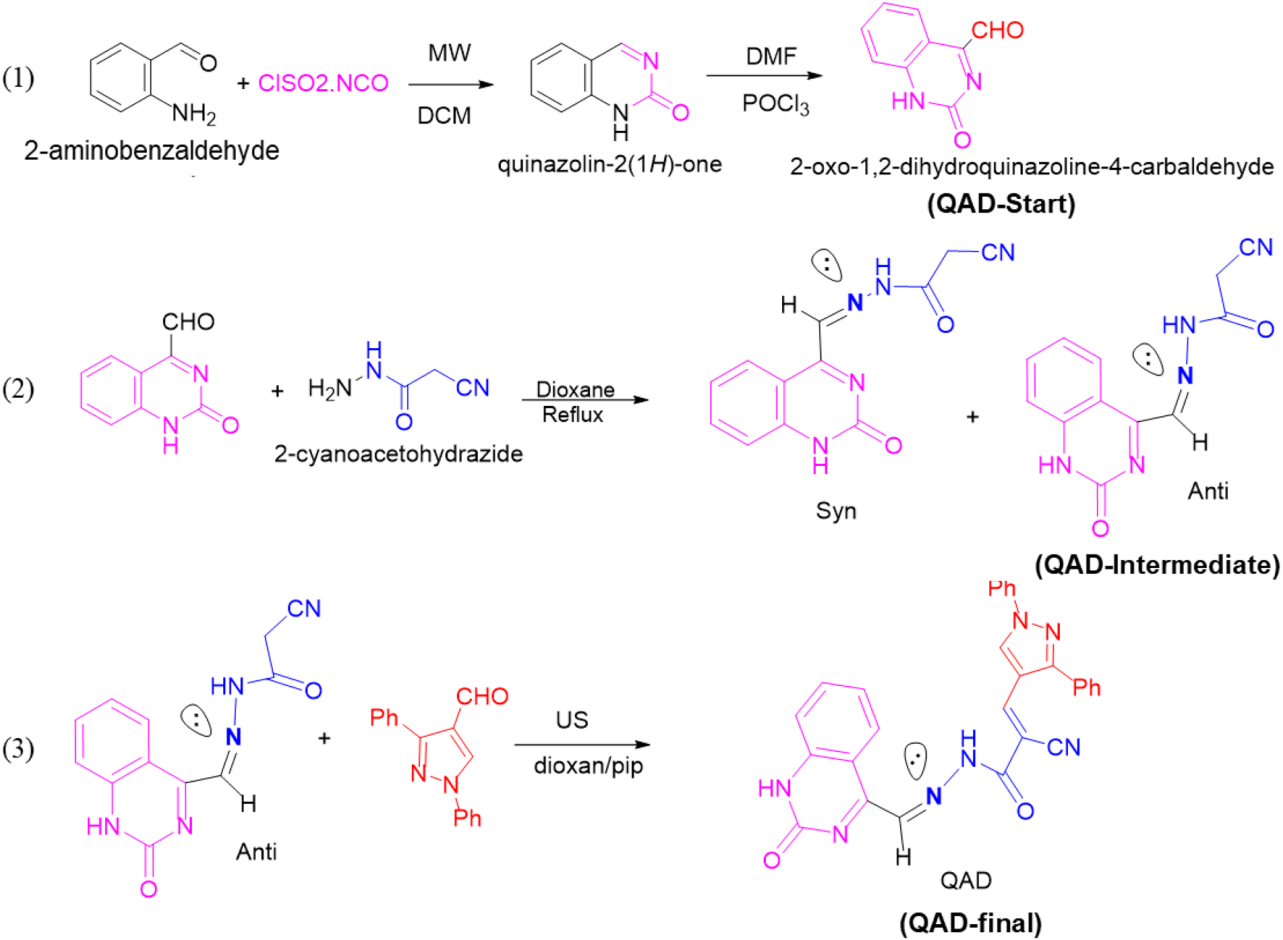
Schematic representation of the synthesis of novel Quinazoline derivative, (*E*)-2-cyano-3-(1,3-diphenyl-1*H*-pyrazol-4-yl)-*N*'-((*E*)-(2-oxo-1,2-dihydroquinazolin-4-yl)methylene)acrylohydrazide, abbreviated as QAD.

The proposed novel Quinazoline-derivative photosensitizer was prepared using a three-step process as illustrated in Fig. 1. In the first step, Quinazolin-2-one can be prepared in a one-pot synthesis starting from commercially available 2-aminobenzaldehyde by its reaction with chlorosulfonyl isocyanate^34^, and Vilsmeier–Haack reaction of the quinazolin-2(1H)-one afforded 2-oxo-1,2-dihydroquinazoline-4-carbaldehyde^35^. In the second step, the anti-isomer of 2-cyano-*N*'-((2-oxo-1,2-dihydroquinazolin-4-yl)methylene)acetohydrazide, (**QAD-Intermediate**) was obtained as follows: a solution of an equimolar mixture of 2-oxo-1,2-dihydroquinazoline-4-carbaldehyde (**QAD-Start**) and 2-cyanoacetohydrazide (0.01 mol) in dioxane (20 mL) was heated under reflux for 2–3 h (TLC); the obtained solid was filtered off and recrystallized from dioxane to afford yellow crystals. In the third step, the obtained yellow crystals of anti-isomer of cyanoacetamide derivative (**QAD-Intermediate**) were reacted as follows: a mixture of cyanoacetamide derivative (0.01 mol) and 1,3-diphenyl-1*H*-pyrazole-4-carbaldehyde (0.01 mol) in 20 mL of dioxane (15 mL) with drops of piperidine under ultrasonic reaction condition (55 °C) at 20 min. Finally, the reaction mixture after cooling was diluted with water and drops HCl and then filtered off, crystallized from ethanol to afford the desired product. The novel synthesized Quinazoline-derivative was named as “(*E*)-2-cyano-3-(1,3-diphenyl-1*H*-pyrazol-4-yl)-*N*'-((*E*)-(2-oxo-1,2-dihydroquinazolin-4-yl)methylene)acrylohydrazide” and abbreviated as the “**QAD-Final**” or “**QAD**”.

### Synthesis of bare TiO_2_ nanoparticles

TiO_2_ nanoparticles were synthesized through a facile template-free sol–gel route, according to our earlier work with some modifications^19,20^. Typically, 50 mL of Titanium tetra-(iso-propoxide) were dissolved in 0.5 L of iso-propyl alcohol then the solutions were magnetically stirred for 1 h. Then, appropriate amounts of distilled water were added dropwise for the conversion of titanium-alkoxide precursor into titanium hydroxide colloidal sol until a milky-like solution was obtained. This solution was magnetically stirred for 2 h, then kept for aging for 2 days to form the TiO_2_ gel. After that, the gel was further purified by filtration and washing numerous times with isopropanol and water, then the powder was collected and dried at 100 ˚C and calcined at 500 ˚C for 5 h. Finally, the obtained powders were denoted as “bare TiO_2_”.

### Synthesis of QAD-sensitized TiO_2_ nanoparticles

The QAD-sensitized TiO_2_ nanoparticles were synthesized through a facile wet impregnation scheme^19,20^. Typically, 1%QAD/TiO_2_ is prepared as follows: 0.05 g of QAD was dissolved in dimethyl sulfoxide (DMSO) and then 5 g of TiO_2_ nanoparticles were added to this solution. Then, the above solution was magnetically stirred for 4 h and left overnight for aging and evaporation of the solvent. Finally, the solids were obtained and further purified through filtration and washing numerous times with methanol and water, and then dried in an electric oven at 80 ˚C, and the as-synthesized sample was denoted as “QAD/TiO_2_”.

### Physicochemical characterizations

Concerning the organic compounds, all melting points were measured on a GALLENKAMP electric melting point apparatus and are uncorrected. ^1^H NMR and ^13^C NMR spectra (δ, ppm) were run at 300 and 75 MHz on a GEMINI NMR spectrometer (GEMINI, Manufacturing & Engineering Inc., Anaheim, CA, USA) utilizing tetramethyl silane (TMS) as an internal standard in deuterated dimethyl sulfoxide. The crystalline parameters of the as-fabricated QAD/TiO_2_ and bare TiO_2_ samples were explored by the X-ray diffraction (XRD) patterns measured by Panalytical X′pert PRO MPD X-ray Diffractometer with Cu Kα radiation (30 mA, 40 kV, λ = 0.15406 nm) in the range of 2θ from 5˚ to 90˚ with a continuous scanning mode, step size of 0.02˚, and step time of 2 s. The morphology images of the as-fabricated materials were investigated using Zeiss SEM Ultra 60 field emission scanning electron microscope (FESEM, 5 kV). The FT-IR spectra were measured by Thermoscientific Pye-Unicam SP-3-300 infrared spectrophotometer using the KBr-discs-based method. The optical absorbance of the as-fabricated materials was studied by measuring the diffuse reflectance spectra (DRS) by Shimadzu UV-2600 UV–Visible spectrophotometer while the measurement mode was absorption in the wavelength range from 200 to 800 nm with a scan step size of 2 nm and barium sulfate was used as a reference sample. The room-temperature photoluminescence (PL) spectra of the as-fabricated QAD/TiO_2_ and bare TiO_2_ samples were measured using a ThermoScientific Lumina fluorometer with the utilization of different excitation wavelengths (300–500 nm).

### Photocatalytic measurements

Aqueous solutions of RB dye were used as a typical representative model of textile dyes as the major organic pollutants to estimate the photocatalytic activities of the as-synthesized QAD/TiO_2_ sample as well as the bare TiO_2_ sample. A commonly used cylindrical batch reactor containing a certain catalyst dose (0.25–3.00 g/L) of the employed catalyst, and 0.1 L of RB (0.5 × 10^–5^–2.0 × 10^–5^ M) solution was employed with adjusting the pH in the range 2–12 using diluted HCl and NaOH solutions. Initially, the reactor was exposed to ultrasonication for 5 min to guarantee the good dispersion of the photocatalysts in the RB solution, then the suspensions were magnetically stirred at 600 rpm for 60 min in the dark (without irradiation) to guarantee the establishment of adsorption–desorption equilibria. Then, the suspensions were exposed to UV irradiation (15W Sylvania UV-A lamp) or visible irradiation (15W Philips Fluorescent lamp) with continued stirring at 600 rpm. Through the irradiation time, 5 mL samples were withdrawn from the suspensions at various time intervals and centrifuged at 2000 rpm for 10 min and the filtrates were separated. Finally, the UV–Visible absorption spectra of the collected filtrates were recorded by Thermoscientific Evolution 300 UV–Visible spectrophotometer, where the RB photodegradation % was assessed from Eq. (1):

1 $$RB \; Photodegradation \; \%= \frac{{A}_{0}- {A}_{t}}{{A}_{0}} \times 100$$

where ‘A_o_ and A_t_’ are the absorbance values of the RB dye at λ_max_ = 554 nm at equilibrium and at a time ‘t’ of irradiation, respectively.

The stability of the as-synthesized QAD/TiO_2_ photocatalyst was investigated by exploring the effect of recyclability of the as-synthesized QAD/TiO_2_ photocatalyst on its photocatalytic activity toward RB photodegradation under UV-A irradiation. The experimental parameters were as follows: [Cat] = 1 g/L, pH  4, [RB] = 1 × 10^–5^ M, dark time = 1 h, UV-irradiation time = 3 h for each cycle. Then, the powder was filtered and washed several times with water and then dried at 60 ˚C. Finally, the samples are re-weighed and dispersed in a fresh RB solution to maintain the same catalyst dose and other conditions are kept constant.

The effects of scavengers were studied to elucidate the reactive species using specific scavengers including methyl iodide (MeI), iso-propyl alcohol (i-PrOH), methyl viologen (MV), and p-benzoquinone (p-BQ) were separately examined. The concentrations of the scavengers were kept constant (1 × 10^–5^ M) upon the comparison while keeping the other parameters to be constant ([Cat] = 1 g/L, pH  4, [RB] = 1 × 10^–5^ M). The effect of oxygen bubbling was studied using the bubbling of oxygen to the solution for 1 h during the dark time only, before the irradiation at the operating conditions ([Cat] = 1 g/L, pH  4, [RB] = 1 × 10^–5^ M).

## Results and discussion

### Characterization of novel Quinazoline-derivative (QAD)

Characterizations of the novel Quinazoline derivatives demonstrated the successful synthesis of the proposed structure named (*E*)-2-cyano-3-(1,3-diphenyl-1*H*-pyrazol-4-yl)-*N*'-((*E*)-(2-oxo-1,2-dihydroquinazolin-4-yl)methylene)acrylohydrazide, abbreviated as QAD. The achieved crystals product structure (QAD) yield 93%. Mp. 324–326 °C, IR: 3424, 3270,3211 (NH), 2209 (C≡N), 1658(C = O), 1604 (C=N). ^1^H NMR (300 MHz, DMSO-*d*_6_): (*anti*-76% and *syn*-isomers 34%) 10.24 (s, 1H, NH, exchangeable by D_2_O), 9.73 (s, 1H, NH, exchangeable by D_2_O), 8.31 (s, 1H, C4-H Quinazoline nucleus), 7.89 (d, 1H, Ar–H, *J* = 7.6 Hz), 7.86 (dd, 1H, Ar–H, *J* = 7.2, 4.2 Hz), 7.53 (d, 1H, Ar–H, *J* = 7.8 Hz), 7.31 (dd, 1H, Ar–H, *J* = 7.2, 4.2 Hz); 5.48 (s, 1H, CH=N), 2.30 and 2.02 (s, 2H, CH_2_ *anti*-76% and *syn*-isomers 34%); ^13^C-NMR, 195, 174, 150, 144, 139, 133, 129, 128, 126, 124, 118, 111, 46, 30, 18. MS, *m/z* (%): 494 (M^+.^, 15.32), 464 (34.21), 453, 385 (54), 332 (50), 325, 308, 247 (25), 165 (35), 94 (36.82), 77 (100). Anal. Calcd. for C_28_H_19_N_7_O_2_ (485.5): C, 69.27; H, 3.94; N, 20.20. found: C, 69.12; H, 3.90; N, 20.13%. The obtained charts are demonstrated in Figs. S1–S5 in the supporting information file.

## Physicochemical characterizations

### Optical absorption of QAD

**Figure 2:**
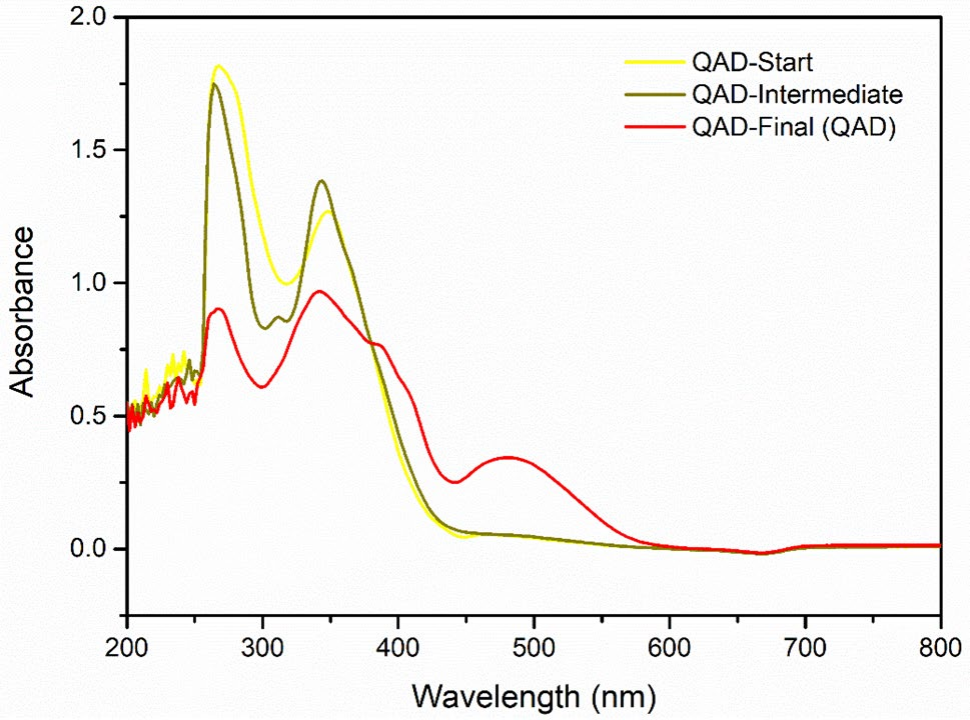
The UV–Visible spectra of the as-fabricated QAD molecules (100 ppm in DMSO): QAD-Start, QAD-Intermediate, and QAD-Final (QAD).

The UV–Visible absorption spectrum of the as-synthesized QAD molecules was compared to the spectra of QAD-Start and QAD-Intermediate samples as shown in Fig. 2. It was found that the QAD-Start and QAD-Intermediate exhibit absorption peaks in the UV region only, around 270 nm and 350 nm, where they demonstrated negligible absorption capability in the visible region. However, the proposed novel structure of QAD-Final (QAD) exhibits the same peaks in the UV region in addition to new 2 shoulder peaks at about 390 nm and 410 nm beside an additional broad absorption peak in the visible range from 440 to 600 nm and centred at about 500 nm. These new peaks and the outstanding peaks of QAD-Final in the visible region can be accredited to the extended conjugations due to the addition of 1,3-diphenylpyrazole-4-carbaldehyde that extended the π-π/n-π conjugation through the aromatic heterocyclic ring and the two phenyl substituents as well as the extended (–N–Ph) and (–C=N–N=C–) bonds added to the QAD-intermediate structure. The significantly extended visible-light absorbance of the proposed QAD molecule indicated the successful synthesis of novel organic dye with visible-light absorption capability. Besides, this suggested the expected outstanding efficiency of QAD as a visible-light photosensitizer to wide bandgap semiconductors in general and specifically for TiO_2_ nanoparticles in this work.

### Crystalline parameters

**Figure 3:**
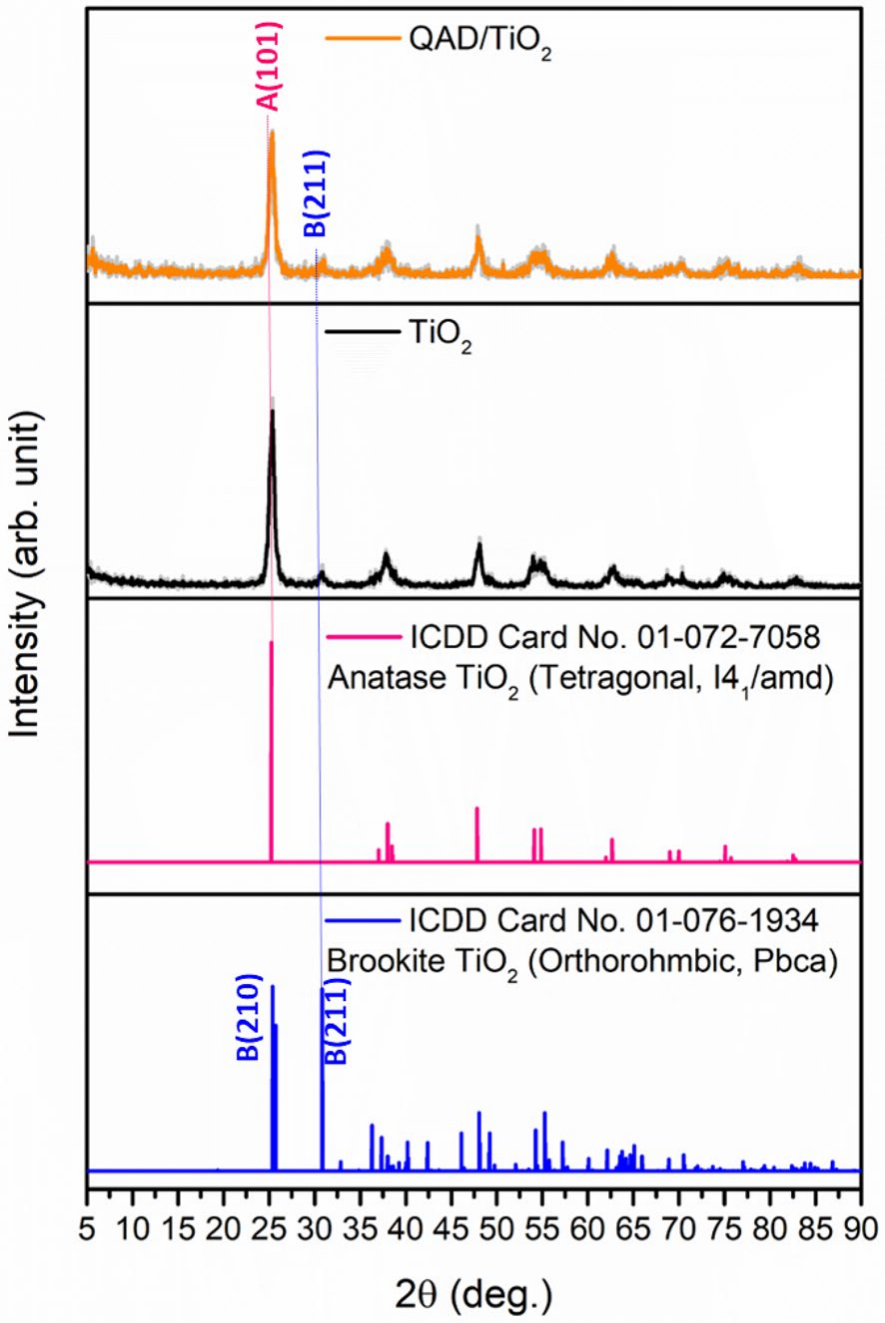
The XRD patterns of the as-synthesized QAD/TiO_2_ and bare TiO_2_ samples.

The crystalline nature of the as-synthesized samples, as well as their crystalline parameters, were investigated in detail to confirm the successful synthesis of TiO_2_ nanoparticles as well as to inspect the influence of the incorporation of QAD molecules on the surface of TiO_2_ nanoparticles. Figure 3 displays the XRD pattern of the as-synthesized QAD/TiO_2_ sample as well as that of the bare TiO_2_ sample. It was found that the XRD patterns of both samples display the existence of the main peaks equivalent to the distinctive planes of the tetragonal anatase phase of TiO_2_ with the I4_1_/amd space group (ICDD Card No: 01-075-2553). The peak observed at 31.06º is characteristic of the (211) plane of the orthorhombic Brookite TiO_2_ with the space group: Pbca (ICDD Card No. 01-075-2549). The relatively low intensity of the peak of (211) plane that should be equivalent to the peak at 25.38 of the (210) plane of Brookite indicates the low percentage of Brookite phase compared to the anatase phase, as stated earlier in our previous work^20^. However, slight shifts in the peaks’ positions were observed upon the incorporation of QAD molecules on the surface of TiO_2_ nanoparticles as shown in Fig. 3, which could be accredited to the impact of the anchoring of the QAD molecules on the lattice structure of TiO_2_ nanoparticles. Besides, the observed change in the relative intensities of the peaks in the QAD/TiO_2_ samples compared to those relative intensities in the bare TiO_2_ sample can indicate the existence of some distortions in the crystalline structure of TiO_2_ after the anchoring of the QAD molecules.

Additionally, the following crystalline parameters including average crystalline size (D, nm) and lattice strain percentage (ɛ, %) at the major plane (101) of tetragonal anatase TiO_2_ were estimated using Eqs. (2) and (3)^8,15,18^:

2 $$D=\frac{k\lambda }{\beta \; cos \; \theta }$$

3 $$\varepsilon =\frac{\beta }{4 \; tan \; \theta }$$

where ‘*k*’ is the shape factor of the nanoparticles and set as 0.9, ‘*λ*’ is the wavelength of the X-rays (λ = 0.15406 nm), ‘*β*’ is full width at half maximum (FWHM) of the diffraction peak, and ‘*θ*’ is the angle of diffraction. It is clearly noticed that the attachment of QAD molecules on the TiO_2_ surface led to a little reduction in the estimated crystalline size in the QAD/TiO_2_ sample when compared to the bare TiO_2_ sample as stated in Table 1.

**Table 1:** The crystalline parameters at the main Anatase plane (101) and the optical bandgap energy of the as-synthesized QAD/TiO_2_ and bare TiO_2_ photocatalysts.

Catalyst	2θ (°)	β (°)	d-spacing (Å)	D (nm)	ɛ (%)	E_g_ (eV)
Bare TiO_2_	25.3008	0.6121	3.51731	13.3	1.190	2.97
QAD/TiO_2_	25.2323	0.6298	3.52964	12.9	1.228	2.58

Furthermore, it was found that the lattice strain% at the (101) plane of TiO_2_ has been slightly increased in the QAD/TiO_2_ sample which can be attributed to the anchoring of the QAD molecules on this plane. Hence, all these findings confirmed that the QAD molecules led to the occurrence of distortions and disorders in the lattice structure of the Anatase TiO_2_ phase. As reported earlier^15,18,36^, it could be assumed that the increase in strain could substantially enhance the electronic and optical properties of the as-fabricated heterojunction in the as-synthesized QAD/TiO_2_ nanoparticles compared to the bare TiO_2_ nanoparticles.

### Structural properties

**Figure 4:**
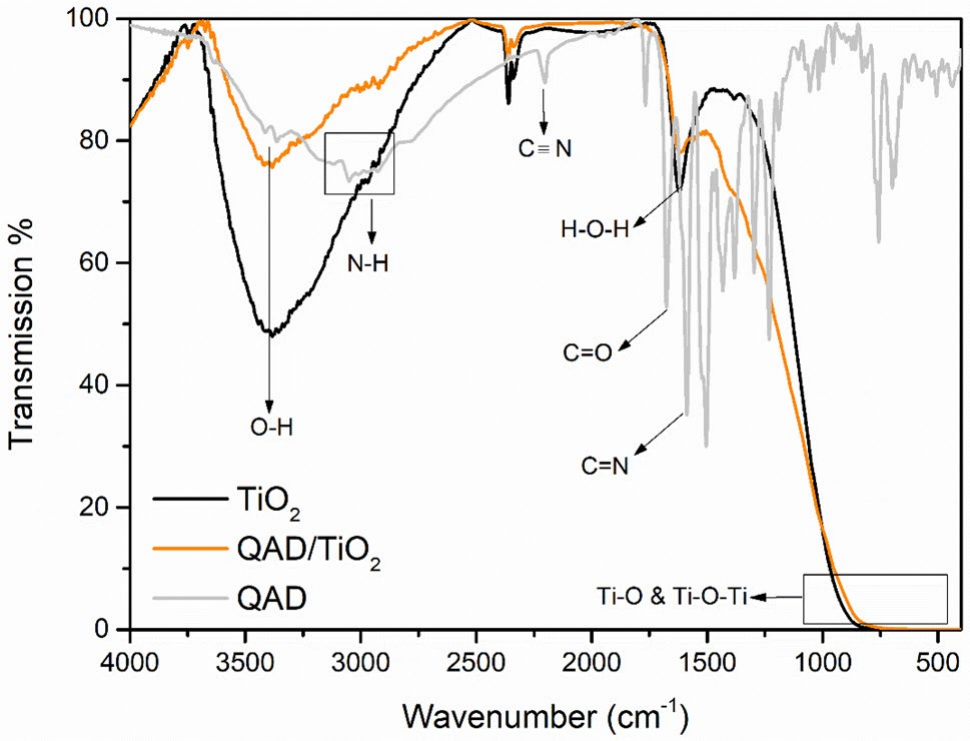
The FTIR spectra of the as-synthesized QAD/TiO_2_ and bare TiO_2_ samples compared to that of the QAD molecules.

The structural characteristics of the fabricated QAD/TiO_2_ heterostructure were investigated by FTIR spectra via the KBr-discs-based method. Figure 4 shows the FTIR spectra of both bare TiO_2_ and QAD/TiO_2_ samples measured in the wavenumber range from 400 to 4000 cm^−1^ to inspect the type of the bonds in the as-synthesized QAD/TiO_2_ sample compared to the bare TiO_2_ sample and to confirm the attachment of the QAD molecules on the surface of TiO_2_ nanoparticles. Concerning the bare TiO_2_ sample, the broad peaks at 3380 and 1620 cm^−1^ can be ascribed to the stretching vibrations of O–H groups and the bending vibrations of H–O–H for the adsorbed water molecules, respectively^15,37^. The broad band in the range from 400 to 820 cm^−1^ could be assigned to those characteristic vibrations of the metal oxide bonds such as the Ti–O–Ti bridging and Ti–O stretching^4^. Similarly, the FTIR spectrum of the as-fabricated QAD/TiO_2_ sample demonstrates both assigned groups of TiO_2_ nanoparticles and QAD molecules with small shifts confirming the successful fabrication of the proposed heterostructure.

### Morphological properties

**Figure 5:**
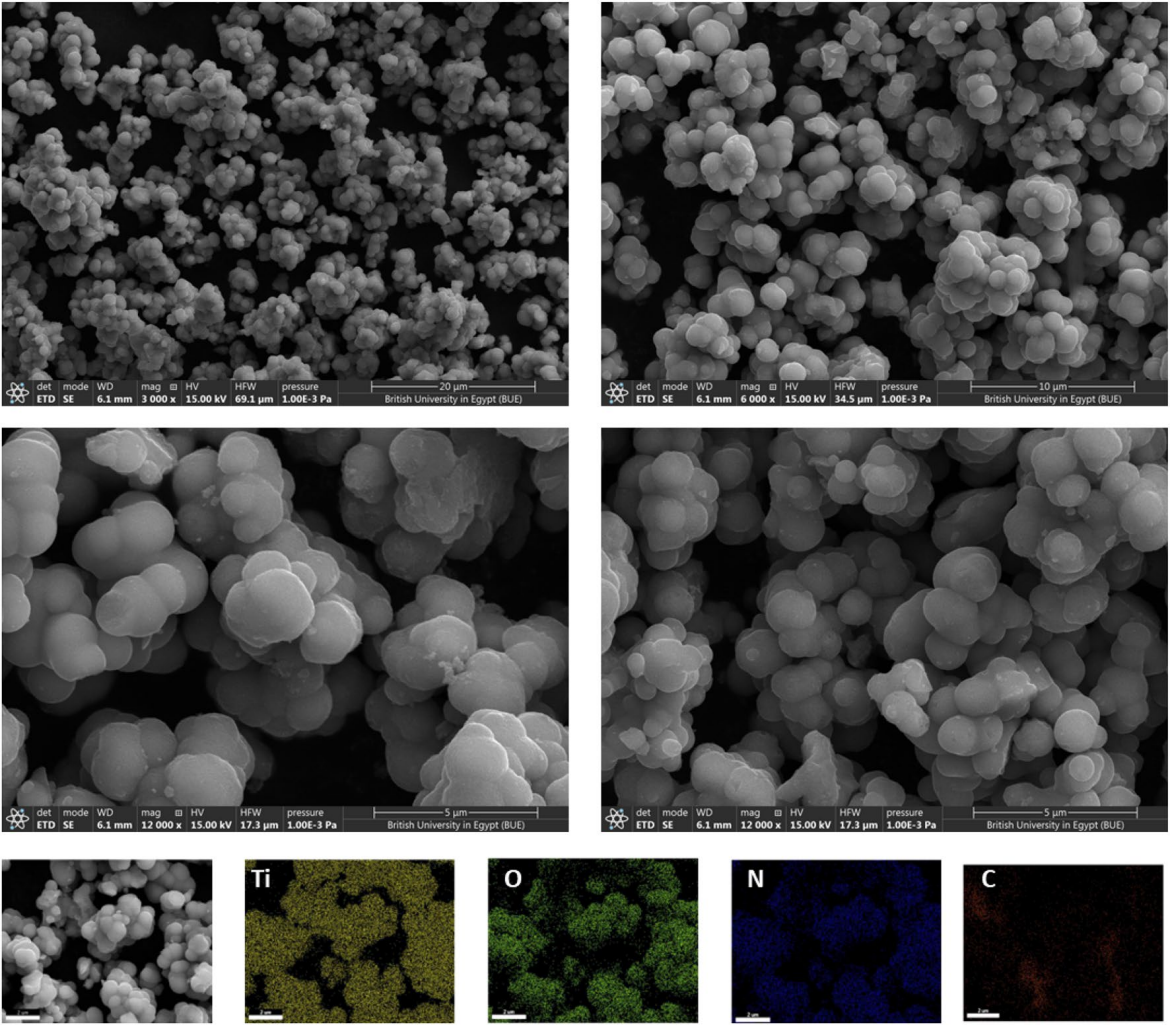
FE-SEM images and the elemental mapping spectra of the QAD/TiO_2_ sample.

The microstructure and the morphology of the as-prepared QAD/TiO_2_ sample have been investigated by FE-SEM as shown in Fig. 5. The FE-SEM images demonstrate the assembly of the TiO_2_ nanoparticles in the form of microsphere morphology and just a few distortions have been observed after the incorporation of QAD molecules on the surface of TiO_2_ microspheres.

As shown in Fig. S6, the EDX spectrum and the corresponding elemental mapping spectra of the bare TiO_2_ sample show the existence of Ti and O elements only among the sample, and the estimated elemental weight and atomic percentages coincide with the typically reported ratios^19^. Besides, the EDX of the QAD/TiO_2_ sample (Fig. S7) shows the presence of N and C elements in addition to the original Ti and O elements after the incorporation of QAD molecules. Additionally, the mapping spectra show the homogenous dispersion of C and N elements among the microspheres of TiO_2_; the lower intensities of C and N elements are attributed to the low percentage of the anchored QAD molecules (~ 1 wt.%). Hence, these findings suggested the success of the employed simple wet-impregnation route to obtain a well-dispersion and homogenous distribution of the organic molecules over the TiO_2_ surface.

### Optical properties 

**Figure 6:**
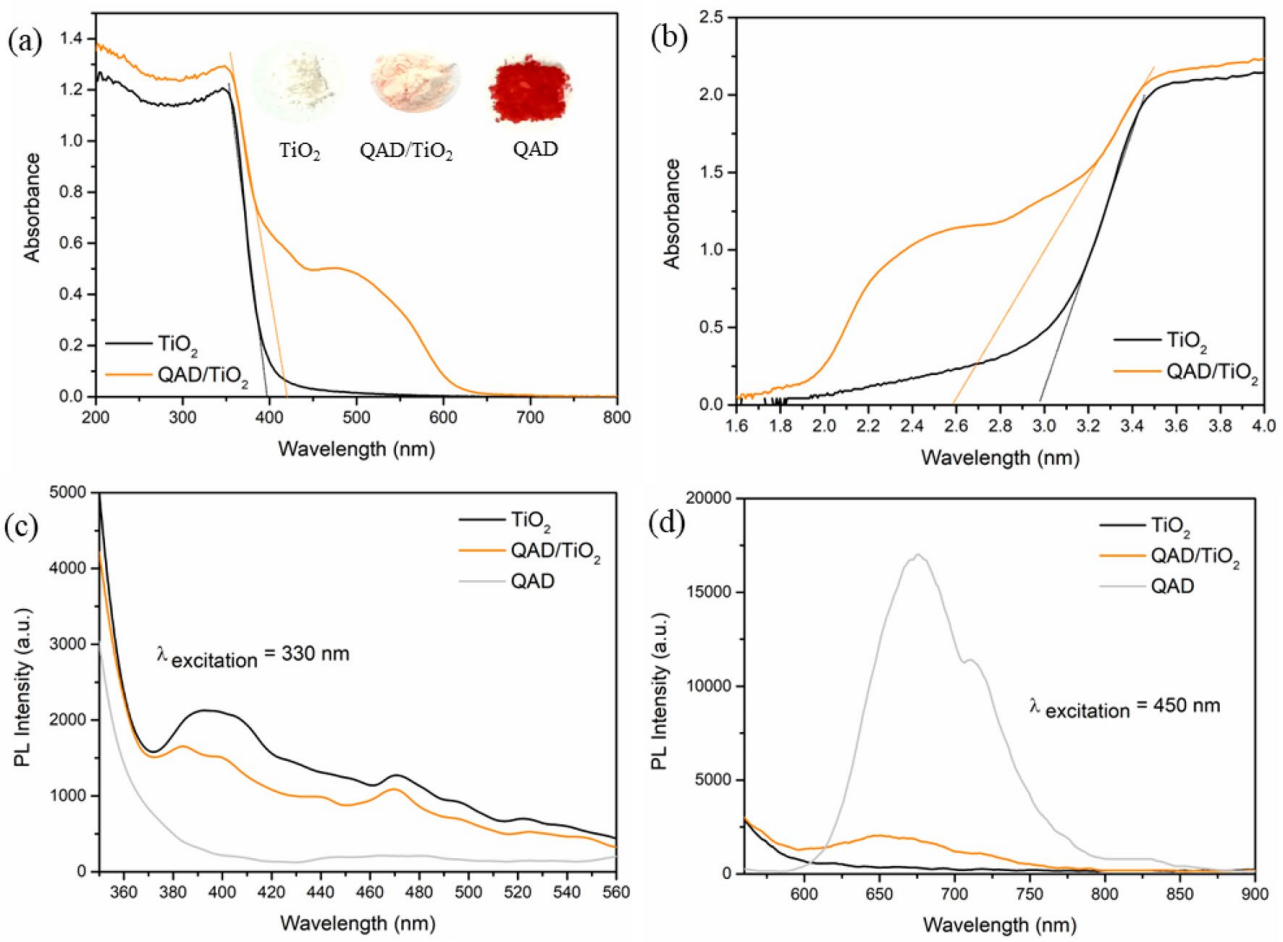
The DRS spectra [inset: photographic images of the powder samples] (**a**), Tauc plots (**b**), and room-temperature PL spectra (**c,d**) of the as-synthesized QAD/TiO_2_ and bare TiO_2_ samples.

The optical properties of the as-synthesized novel QAD/TiO_2_ heterostructure were studied by the inspection of its absorption profile and the estimation of its bandgap energy (E_g_) and compared to those of the bare TiO_2_ nanoparticles as well as investigating the photoluminescence (PL) data to evaluate the e–h recombination rates. Figure 6a showed the enhanced visible absorption of the QAD/TiO_2_ sample compared to the bare TiO_2_ sample, confirming the successful role of the incorporated QAD molecules as visible-light photosensitizer. Likewise, the assembled Tauc plots (Fig. 6b) revealed that the assessed bandgap energy of bare TiO_2_ is 2.97 ± 0.05 eV which has been significantly decreased to 2.58 ± 0.05 eV after the incorporation of QAD molecules. Additionally, the anchoring of QAD on the TiO_2_ surface not only did a remarkable shift in the absorbance edge of TiO_2_, but it also created an absorption tail and a wide absorption peak in the visible range from 400 to 650 nm. This confirmed the successful role of QAD as an outstanding visible antenna for TiO_2_ to improve its light absorption capability in the visible light zone.

Photoluminescence (PL) is one of the potent techniques utilized for studying the optical properties of the semiconductors, especially their e–h recombination rates as well as their capabilities for charge separation, whereas the emission peaks are attributed to the recombination of the photoinduced electrons in the conduction band (CB) with the photoinduced holes in the valence band (VB)^17^. Figure 6c demonstrates the PL spectra of the as-synthesized materials measured at room temperature with λ_ex._ of 330 nm, where the bare TiO_2_ sample demonstrated the highest PL intensity demonstrating their relatively fast e–h recombination rates. Nevertheless, the QAD/TiO_2_ sample showed a remarkable reduction in the PL intensity proposing that the anchoring of QAD molecules on the TiO_2_ surface could effectively achieve a drop in the recombination rate and enhancement in the charge separation rather than that of the bare TiO_2_ nanoparticles. Besides, Fig. 6d depicts that the PL intensity of the QAD/TiO_2_ is much lower than that of QAD confirming that QAD molecules and TiO_2_ enhance the lifetime in the excited state of each other through the charge transfer and injection of electrons from the QAD molecules to the TiO_2_ nanoparticles as described in detail in the mechanism section (Fig. 9). It is essential to confirm that the same trend and the same findings have been revealed upon the use of different excitation wavelengths as demonstrated in Fig. S8. Hence, we expect that the as-prepared QAD/TiO_2_ nanoparticles would exhibit a significant enhancement in the photocatalytic performance rather than that of the bare TiO_2_ nanoparticles.

## The photocatalytic performance of QAD/TiO_2_ nanoparticles

### Control tests

**Figure 7:**
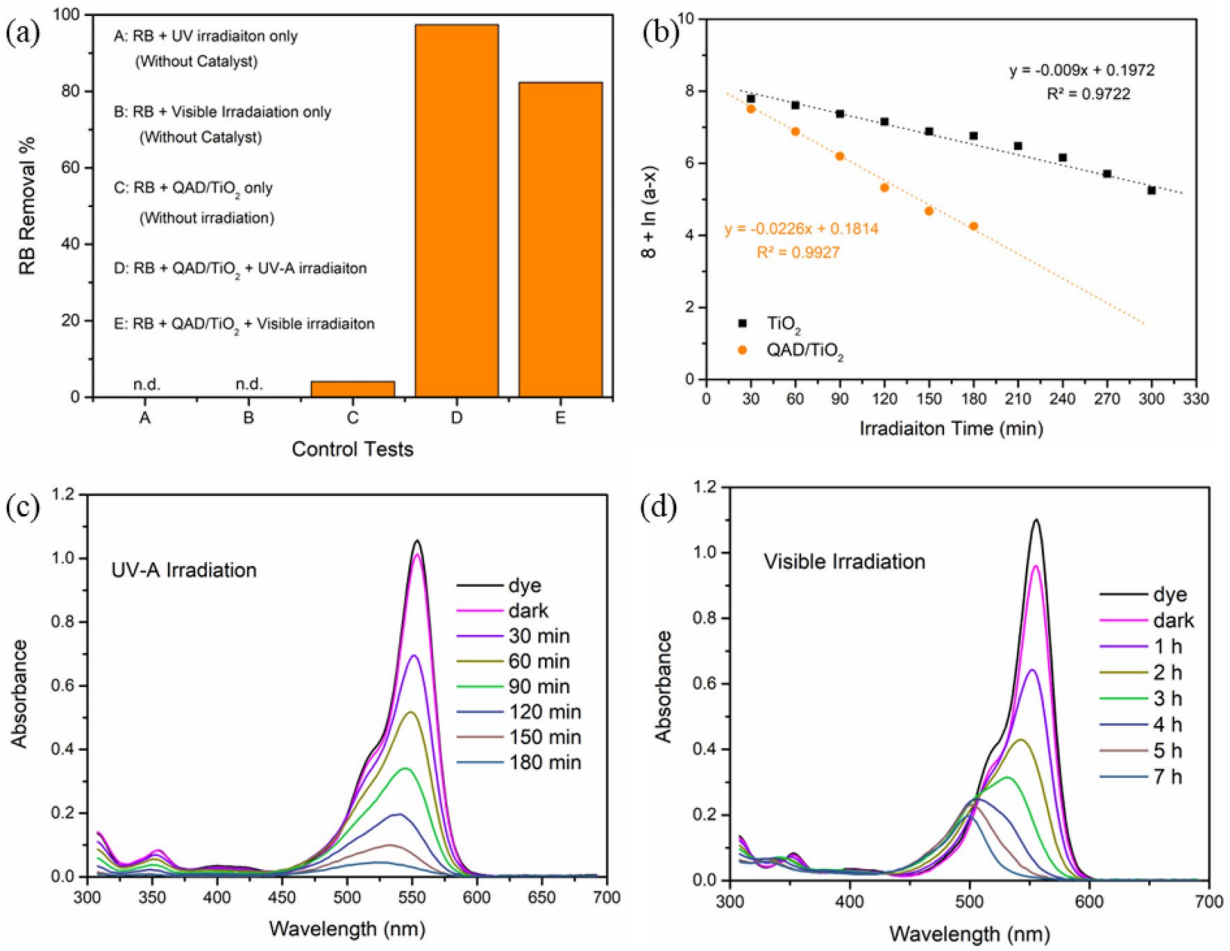
The control testes (**a**), the first-order plots of the photodegradation of RB using bare TiO_2_ and QAD/TiO_2_ (**b**), the temporal changes in RB UV–Visible spectra using the QAD/TiO_2_ nanoparticles under the irradiation of different irradiation sources: UV-A irradiation (**c**) and Visible light irradiation (**d**) (experimental parameters: [Cat] = 1 g/L, pH  6, [RB]_o_ = 1 × 10^–5^ M, dark time = 1 h, UV-irradiation time = 180 min, Visible irradiation time = 7 h).

Aqueous solutions of RB dye were used as a representative simulated wastewater sample including a model of organic water contaminants to measure the photocatalytic activities of QAD/TiO_2_ under UV-A/Visible irradiations. First, the control experiments including the degradation of RB via photolysis under UV-A/Visible irradiations (without catalyst) and the removal of RB dye in the dark via adsorption on the catalyst surface (without irradiation) were studied as shown in Fig. 7a. It was found that there were no changes in the RB absorbance were detected in the control experiments, confirming the photostability of the RB dye against photolysis under UV-A/Visible irradiations in addition to the insignificant role of the adsorption route. On the other hand, the exposure of the suspensions of QAD/TiO_2_ and RB solution to UV-A/Visible irradiations achieved a significant drop in the absorbance of RB and its complete photodegradation signifying that the photodegradation process is attributed to the combination of both QAD/TiO_2_ nanoparticles dispersed in RB solution and the UV-A/Visible irradiations; these results coincide with the earlier stated findings^17^.

### QAD/TiO_2_ vs bare TiO_2_

The incorporation of QAD molecules on the TiO_2_ surface caused a double-fold enhancement in the photocatalytic activity of QAD/TiO_2_ nano-heterostructure compared to the bare TiO_2_ nanoparticles, where QAD/TiO_2_ achieved almost complete photodegradation (~ 98%) of RB dye in 3 h only under UV-A irradiation. However, 5 h were required to achieve 93% degradation of RB using the bare TiO_2_ sample while maintaining the other parameters (catalyst dose ([Cat]), dye concentration, pH, irradiation source, etc.) to be constant. Besides, the photodegradation process was found to follow first order kinetics as reported earlier^17,18^, and the estimated observed rate constants (*k*_*obs*_) were found to be 0.0226 min^−1^ and 0.0090 min^−1^ for QAD/TiO_2_ and bare TiO_2_, respectively (Fig. 7b); which means the *k*_*obs*_ has been increased by about threefold enhancement, at the same conditions, due to the incorporation of QAD photosensitizer. Therefore, these results showed the outstanding effect of incorporation of a small amount of QAD molecules (less than 1%) on the TiO_2_ surface to enhance their photocatalytic activity towards the photodegradation process and boost the kinetics of the reaction, which could be ascribed to the enhancing of the charge separation as suggested from the PL data mentioned above. Thus, starting from this point, in the following sections our story will focus on the proposed novel QAD/TiO_2_ nanoparticles and the optimization of the parameters affecting their photocatalytic performance toward RB photodegradation and elucidating the mechanism of enhancement of TiO_2_ by novel QAD photosensitizer.

### UV and Visible photoactivity of QAD/TiO_2_

The photocatalytic activity of the as-fabricated QAD/TiO_2_ heterojunction was investigated under visible-light irradiation to investigate their photocatalytic behaviour under visible irradiation compared to UV-A irradiation and to show whether the type of irradiation influences the degradation pathway or not. Figure 7c demonstrates the decline in the absorbance of RB at 554 nm, which is characteristic of the “n → π transitions” in the bathochromic bonds (C=N and C=O) in the structure of RB; this is an indication of the degradation of these bonds which caused the decolourization of the dye solution. In addition to this, the temporal broadening and vanishing of the peaks in the UV range confirmed the complete photodegradation of the nuclei of the aromatic rings in the RB dye, representing the entire mineralization of RB into H_2_O and CO_2_^17,38,39^.

On the other hand, upon investigating the photocatalytic performance of QAD/TiO_2_ under visible irradiation (Fig. 7d), it was found that the RB removal% was found to be about 82% after 3 h and the observed rate constant has been reduced to 0.0146 min^−1^ under Visible irradiation compared to the UV-A irradiation at the same parameter, as shown in Fig. S9. This reduction in the observed RB removal% could be assigned to the fact of the higher energy of UV-A photons compared to the lower energy of the visible photons^14,20^. However, it was found that there are significant gradual blue shifts in λ_max_ of RB dye altered from 554 to 498 nm after 7 h of exposure to visible irradiation under a fluorescent lamp. These blue shifts can be attributed to the N-deethylation of the amino groups in the RB structure, as stated earlier in the literature^16,20,40^. Thus, the mineralization of RB dye cannot be achieved under visible light irradiation due to the nature of the RB dye itself and the different mechanisms of its degradation under visible light. The above-mentioned findings revealed that the type of irradiation not only can influence the photodegradation% and observed rate constant, but it also affects the reaction pathway as well as the degradation mechanism.

### Factors affecting the photodegradation process

In general, there are many factors affecting the photodegradation processes starting from the nature of the photocatalyst, the nature of the pollutant itself (its chemical structure, being cationic or anionic, etc.), irradiation type (UV-A, UV-C, Visible, etc.), light source power, the distance between the reactor and the light source, reactor diameter, catalyst dose ([Cat]), initial pollutant concentration ([RB]_0_), pH, etc. Herein, we are performing our experiments using the same photocatalyst (QAD/TiO_2_ nanoparticles), the same organic pollutant (RB dye), and the same light source (15 W UV-A irradiation where the distance between the reactor and the lamp was maintained to be 10 cm), and the same reactor diameter (10 cm). Thus, the three factors that are going to be optimized and investigated in the following subsections are the parameters of pH, catalyst dose, and initial dye concentration, as listed in Table 2.

**Table 2:** The influence of different factors affecting the photocatalytic activity of the as-fabricated QAD/TiO_2_ toward RB photodegradation in UV-A irradiation.

pH	[Cat] (g/L)	[RB]_o_ (× 10^–5^ M)	*k*_*obs*_ (× 10^–4^ min^−1^)	R^2^
2	1.00	1.0	96	0.9986
4			306	0.9772
6			223	0.9736
8			226	0.9649
10			177	0.9878
12			73	0.9928
4	0.25	1.0	80	0.9944
	0.50		225	0.9761
	1.00		281	0.9927
	1.50		338	0.9786
	2.00		404	0.9923
	3.00		365	0.9807
4	1.00	0.5	378	0.9885
		1.0	226	0.9927
		1.5	134	0.9967
		2.0	116	0.9966

### Effect of pH

Usually, the effluents of textile wastewater exhibit wide-ranging pH values, and it was reported that the pH parameter can play critical roles in wastewater treatment owing to its effects on (1) the features of effluents of wastewater, (2) the adsorption of the pollutants on the surface of the photocatalysts/adsorbents, and (3) the photo-induced production of reactive species^41^. Thus, the pH is considered one of the critical parameters influencing the interactions of the organic dye with the catalyst surface and accordingly affecting the photodegradation rates^19^. The change in the pH of the solution would adjust the charge of the catalyst surface; thus, we estimated the point of zero charges (PZC) of the as-fabricated QAD/TiO_2_ photocatalyst using the facile pH drift method^16,42^. Figure S10 showed that the PZC of the as-synthesized QAD/TiO_2_ exhibit a pH_PZC_ = 5, revealing that the surface charge of the QAD/TiO_2_ will be negative at pH > 5 and positive at pH < 5, which is in good accordance with the reported PZC range of TiO_2_ in the literature^43^. Then, the effect of pH has been investigated in the range of pH 2–12 under UV-A irradiation, as shown in Fig. S11, while other factors remained constant; the observed rate constants (*k*_*obs*_) and the corresponding correlation factors (R^2^) are recorded in Table 2. It is clearly observed that the rate of RB photodegradation was reduced by increasing the pH above 4 in the range of 6–12; this can be attributed to the repulsion between the negatively deprotonated COO^-^ group (pK_a_ = 3.7) and negative QAD/TiO_2_ catalyst (pH_PZC_ = 5) at this pH range. Besides, another valid reason for this decrease in the photodegradation rates is the fact of the formation of RB zwitterions and their aggregation^19,44^. The formation of these zwitterions can be attributed to the attractive electrostatic interaction between the negatively charged carboxyl groups (–COO^−^) and the positively charged amino groups (–N^+^) in the RB monomers, and consequently forming a dimer structure with a larger molecular form which hinders its interaction with the active sites of QAD/TiO_2_ photocatalyst^19,44^. On the other hand, at pH  2, both RB and QAD/TiO_2_ are positively charged; thus, there would be an electrostatic repulsion between them, which reduces the photodegradation rates at very low pH values. However, the optimum pH value was pH  4, where there will be an attraction between the positively charged QAD/TiO_2_ surface and the negatively charged RB molecules, which facilitates the approach of the RB molecules toward the active sites of photodegradation on the as-synthesized QAD/TiO_2_ sample. Finally, it is important to state that there are no peaks corresponding to the QAD molecules have been observed in the UV–Visible spectra even at higher pH values indicating the stability of the QAD molecules under harsh conditions^19^. Hence, the following photodegradation experiments will be operated at the optimized pH value of 4.

### Effect of catalyst dose

The effect of the photocatalyst doses can be considered one of the crucial parameters influencing the photodegradation processes to achieve the best efficiency relative to the employed catalyst loading. Thus, the photocatalytic performance of QAD/TiO_2_ nanoparticles was studied under UV-A irradiation using different loadings from 0.25 to 3.00 g/L, as shown in Fig. S12; while other factors remained constant: pH  4 and [RB] = 1 × 10^–5^ M. At the low loadings range (0.25–0.50 g/L), the observed rate constant was significantly increased by about threefold upon doubling the photocatalyst dose from 0.25 to 0.50 g/L, as recorded in Table 2. This can be attributed to the increase of the available active sites correlated to the loading amount of the catalyst with respect to the same amount of the target dye (RB). However, the observed rate constants and the corresponding photodegradation percentages are gradually increased during the subsequent increases of the catalyst dose from 0.50 to 2.00 g/L, as shown in Fig. S13. Besides, there was a decrease in the *k*_*obs*_ constant and the corresponding RB photodegradation% after the increase of the photocatalyst dose from 2.00 to 3.00 g/L; this could be ascribed to the screening effect and the scattering of light caused by the excess QAD/TiO_2_ nanoparticles which imped of the light penetration to other active sites^17,19,45^. Hence, the optimum catalyst dose range is 0.50–1.00 g/L; and any increase in the catalyst doses not only can be considered a “dead mass”, but also it has a negative influence on the photocatalytic activity of the as-synthesized QAD/TiO_2_ photocatalyst.

### Effect of initial RB concentration

The effect of the initial RB concentration was studied in the range of absorbance that obeys Beer-Lambert law with an absorption value below 2; thus, the suitable range of [RB] is from 0.5 × 10^–5^ to 2 × 10^–5^ M, while the other factors were maintained unchanged. As shown in Table 2 and Fig. S14, the increase of the [RB]_0_ caused the decrease of the RB photodegradation% and the *k*_*obs*_ constant was decreased accordingly. This behaviour is commonly noticed because of the reduction in the number of photons reaching the surface of the photocatalyst to photo-induce the generation of the reactive species because the high concentrations of RB dye behave as an inner-filter solution for the photons in addition to its blocking for more active sites of the catalyst compared to the case in the lower RB concentrations^17,19^. This is one of the general obstacles of all advanced oxidation processes (AOPs) and the practical/industrial application will require large facilities for dilution of the pollutant concentration to avoid a decrease in the activity; however, the other advantages of the AOPs outweigh this obstacle.

### Stability of QAD/TiO_2_ photocatalysts

**Figure 8:**
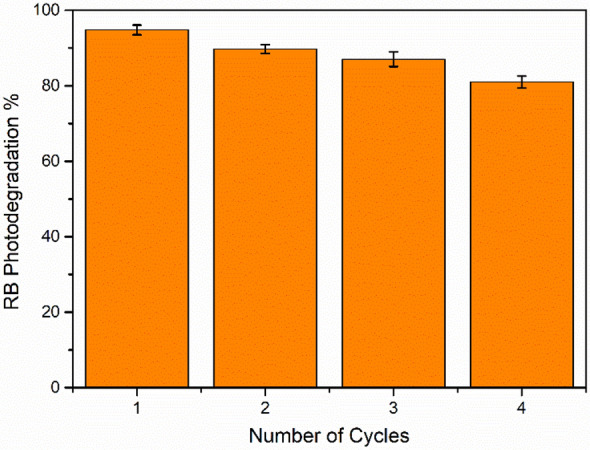
The effect of recyclability of the as-synthesized QAD/TiO_2_ photocatalyst on its photocatalytic activity toward RB photodegradation under UV-A irradiation (experimental parameters: [Cat] = 1 g/L, pH  4, [RB] = 1 × 10^–5^ M, dark time = 1 h, UV-irradiation time = 3 h for each cycle).

One of the major obstacles to the practical application of organic dye sensitizers is the stability of the QAD molecules in the presence of highly reactive radicals and oxidative species. The stability of the as-fabricated QAD/TiO_2_ sample was investigated over four consecutive cycles where the powder was washed after each cycle with distilled water numerous times and finally dried in an electric oven at 60 °C and re-employed in the next cycle with fresh RB dye solution with maintaining the same operating parameters. It was noticed that the photocatalytic activity was in the same range after four consecutive cycles as displayed in Fig. 8; the standard error calculations are demonstrated in Table S1. These findings signifying the stability of the as-fabricated QAD/TiO_2_ photocatalyst that could be utilized in water treatment and reused for various cycles. Hence, all the above findings revealed the stability of the proposed photocatalyst.

## Mechanism of the enhanced performance of QAD/TiO_2_

### Effect of scavengers

Generally, TiO_2_ is a semiconductor composed of VB and CB with a bandgap energy of 2.97 eV, when the TiO_2_ is absorbed by a photon with energy exceeding 2.97 eV, the electrons are transferred from the VB to the CB, leaving behind positive holes in the VB. Then there are four possibilities for the degradation of RB: (1) oxidation of RB molecules through the reaction of RB with ${h}_{VB}^{+}$, (2) oxidation of the RB molecules using the OH^**∙**^ produced upon the reaction of ${h}_{VB}^{+}$ with H_2_O molecules, (3) reduction of RB molecules by the photoinduced electrons (${e}_{CB}^{-}$), and (4) the reduction of RB molecules using ${\mathrm{O}}_{2}^{\cdot -}$ produced upon the reaction of ${e}_{CB}^{-}$ with the dissolved O_2_.

**Figure 9:**
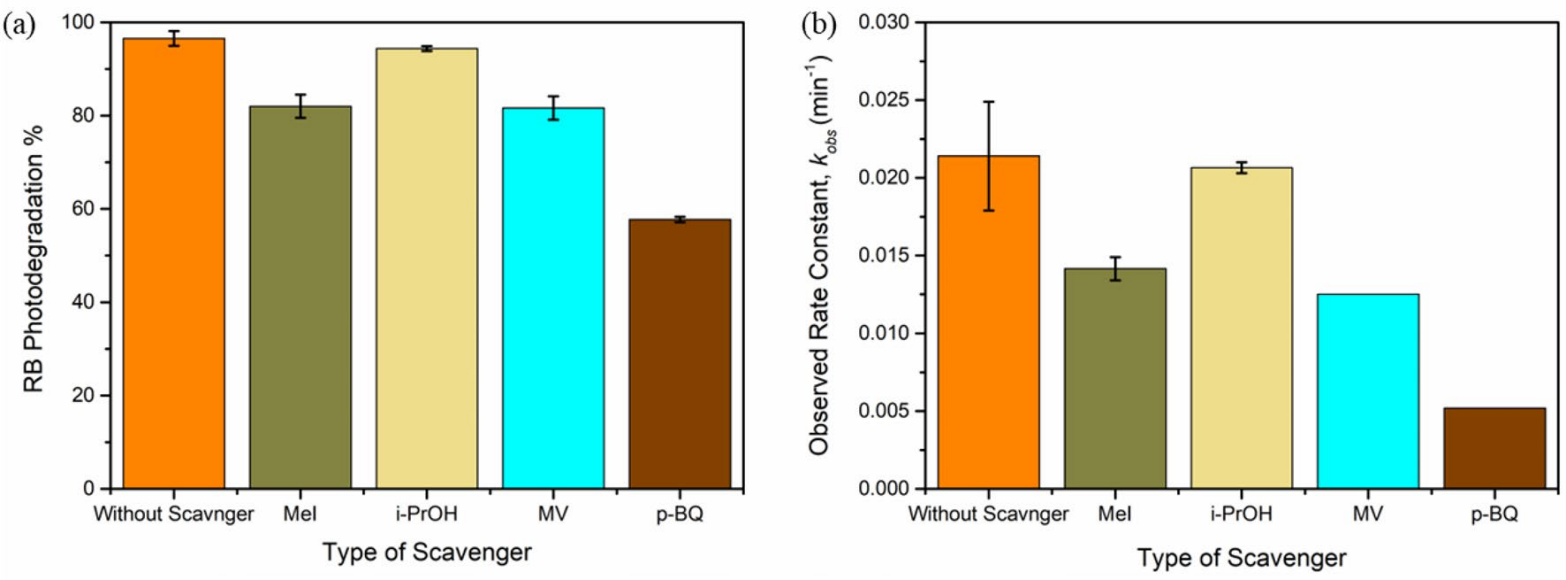
The effect of scavengers on the RB photodegradation% in 150 min (**a**) and the corresponding 1st order plots (**b**) using QAD/TiO_2_ photocatalyst under UV-A irradiation (experimental parameters: [Cat] = 1 g/L, pH  4, [RB] = 1 × 10^–5^ M, [Scavenger] = 1 × 10^–5^ M).

The influence of the types of different scavengers on the photocatalytic activity of QAD/TiO_2_ nanoparticles was studied to evaluate the most reactive species involved in the photodegradation process. Thus, methyl iodide (MeI), iso-propyl alcohol (i-PrOH), methyl viologen (MV), and p-benzoquinone (p-BQ) were separately examined as specific scavengers for the trap of ${h}_{VB}^{+}$, OH^**∙**^, ${e}_{CB}^{-}$, and ${\mathrm{O}}_{2}^{\cdot -}$, respectively^16,19,46^. The concentrations of the scavengers were kept constant (1 × 10^–5^ M) upon the comparison, as displayed in Fig. 9a, while keeping the other parameters to be constant ([Cat] = 1 g/L, pH  4, [RB] = 1 × 10^–5^ M). The standard error calculations are demonstrated in Table S2. It was found that the lowest activity was detected in the presence of p-BQ indicating the ${\mathrm{O}}_{2}^{\cdot -}$ radicals were the most producible reactive species in the photodegradation of RB using the as-fabricated QAD/TiO_2_ nanoparticles, as reported earlier in similar works^19,24^.

Moreover, to further confirm that the ${\mathrm{O}}_{2}^{\cdot -}$ radicals are the most reactive species, and the effect of O_2_ bubbling on the photocatalytic performance of the QAD/TiO_2_ photocatalyst was studied. It was noticed that the bubbling of O_2_ gas to the photocatalyst/RB suspensions during the dark time achieved a significant enhancement in the observed rate constant by about 70% (Fig. S15) confirming that the dissolved O_2_ is participating in the RB photodegradation process. This double enhancement can be attributed to the increase in the dissolved O_2_ concentration due to the O_2_ bubbling process, which facilitates the formation of ${\mathrm{O}}_{2}^{\cdot -}$ radicals and consequently increase the photocatalytic performance. Hence, this supported that the ${\mathrm{O}}_{2}^{\cdot -}$ radicals were the most reactive radicals in the photodegradation process using the as-fabricated QAD/TiO_2_ nanoparticles under UV-A irradiation.

### The proposed mechanism

**Figure 10:**
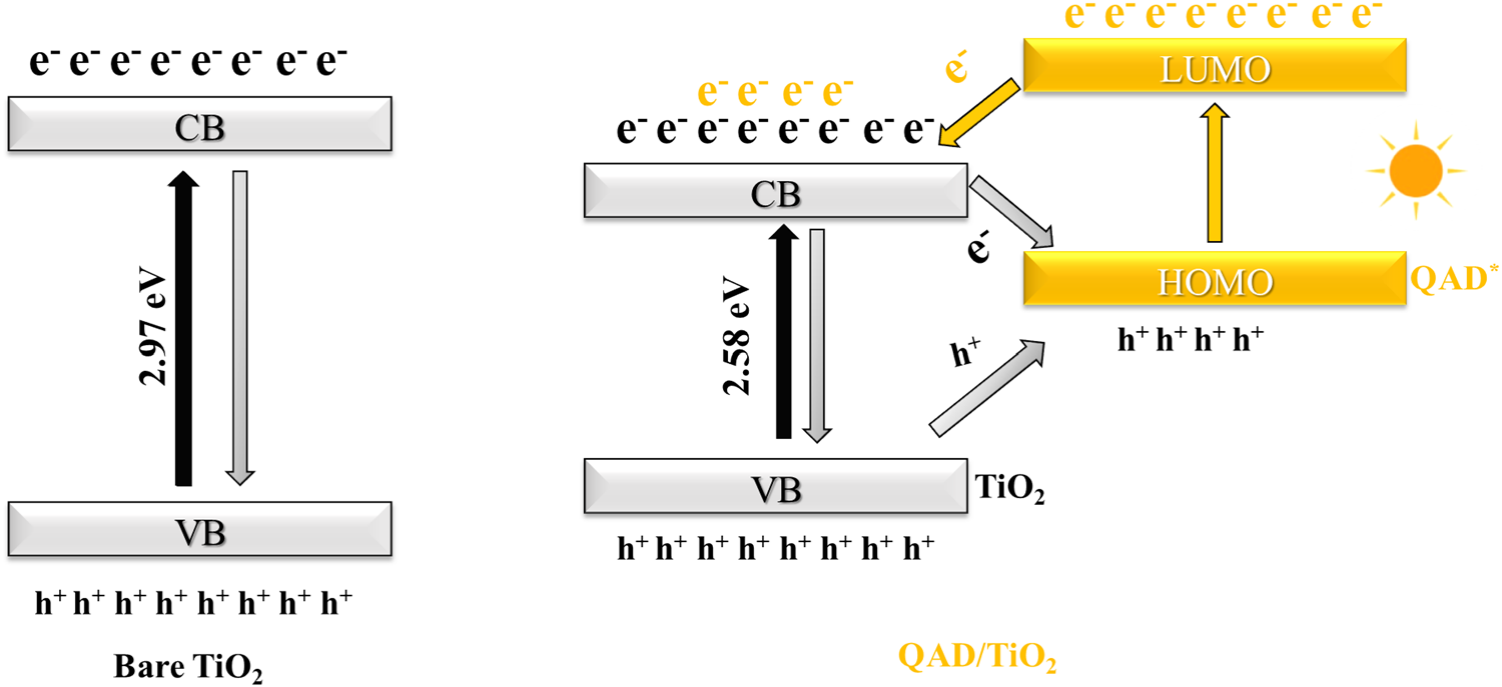
The typical mechanism of the bare TiO_2_ photocatalysts and the proposed mechanism of the enhanced photocatalytic performance of QAD/TiO_2_ based on the enhancement of the charge separation as supported by PL results. (The bandgap energies are deduced from the DRS findings, and the relative positions of QAD’s HOMO/LUMO levels compared to TiO_2_^’^s VB/CB are proposed based on the PL findings).

According to the effect of scavenges on the RB photodegradation and the above-mentioned PL and DRS data, the mechanism of the QAD/TiO_2_ photocatalyst was suggested and associated with that of the bare TiO_2_ sample, as shown in Fig. 10. In the bare TiO_2_ sample, after the excitation of the sample by highly energetic photons with energy exceeding 2.97 eV; i.e. UV photons only, the e–h pairs are formed and the e–h recombination is the most predominant pathway. However, upon the exposure of the QAD/TiO_2_ photocatalyst exposed to the light photons, the TiO_2_ with narrower bandgap energy (2.58 ± 0.05 eV) can be excited by the visible-light photons and the QAD molecules anchored on the TiO_2_ surface are excited to be QAD^*^ where the electrons can transfer from the highest occupied molecular orbital (HOMO) to the lowest unoccupied molecular orbital (LUMO) of QAD. Then, the photoexcited electrons from QAD^*^ can be introduced into the CB of TiO_2_ beside the photoinduced ${e}_{CB}^{-}$ which leads to the increase of the concentration of electrons in the CB of TiO_2_; this assumption of injection is supported by the reduction of PL intensity of QAD in the QAD/TiO_2_ compared to the bare QAD sample. Subsequently, the high concentration of the reactive photoinduced electrons in the CB of QAD/TiO_2_ increased the probability of the reduction of the dissolved O_2_ molecules to form O_2_^.−^ radicals, which are responsible for the photodegradation of RB via a reductive degradation pathway, as supported by the results mentioned above regarding the effects of scavengers and O_2_ bubbling.

Moreover, the photoinduced positive holes on the VB of the excited TiO_2_ can spontaneously transfer to the HOMO of QAD leading to the reduction of the tendency of the excited electrons in the CB to return to the VB. Alternatively, the electrons have a new level, HOMO of QAD, which is available for relaxation rather than the original VB of TiO_2_ leading to the increase in the probability of availability of oxidative species to degrade the RB molecules. Hence, the as-fabricated heterojunction upon the anchoring of QAD molecules on the surface of TiO_2_ provided different pathways for enhanced charge separation rather than the recombination route as evidenced by the reduction of PL of QAD/TiO_2_ when compared to the bare TiO_2_ and pure QAD samples. This mechanism can interpret the enhanced optical properties and the improved photocatalytic activity of the as-synthesized QAD/TiO_2_ compared to the bare TiO_2_ nanoparticles signifying the outstanding role of the efficient proposed structure of QAD as a novel organic dye sensitizer.

### Future outlook

The findings and outputs of this research article state that the efficient photosensitization of TiO_2_ using the novel QAD molecules confirmed their role in enhancing the optical properties of TiO_2_ by decreasing its band gap energy, reducing the e–h recombination rates, and creating a visible-light antenna to extend its activity to the visible region. Hence, these findings will open the door for investigating numerous organic sensitizers based on Quinazoline derivatives and other heterocyclic compounds by tuning the heterocyclic structure via various cycloaddition reactions to further extending of the light absorption capability of the organic photosensitizers toward the visible region. Finally, the proposed QAD/TiO_2_ samples are expected to exhibit exceptional photocatalytic performance when utilized in the photodegradation of further organic water pollutants, other photocatalytic applications, and/or antibacterial applications.

## Conclusion

A novel organic compound (Quinazoline-derivative, QAD) has been synthesized to be used as a novel organic dye sensitizer for TiO_2_ nanoparticles. The FTIR, NMR, and UV–Visible spectra confirmed the successful synthesis of the proposed structure of novel QAD. Anatase-phase TiO_2_ nanoparticles had been synthesized through a simple template-free sol–gel approach with a microsphere morphology as suggested by the XRD and SEM techniques. The as-synthesized novel QAD photosensitizer was anchored on the surface of TiO_2_ and made slight distortions in the crystal structures, a remarkable reduction in the bandgap energy of its band gap energy to 2.58 ± 0.05 eV, and sufficient reduction of the e–h recombination rates. Consequently, the photocatalytic activity of the as-fabricated QAD/TiO_2_ sample has been enhanced and *k*_*obs*_ increased by about 3-folds towards the photodegradation of RB dye. The effects of the scavengers and oxygen-bubbling experiments suggested that the superoxide anion radicals (${\mathrm{O}}_{2}^{\cdot -}$) were the most reactive radicals in a reductive degradation pathway. Finally, the mechanism of the enhancement of the photocatalytic performance of QAD/TiO_2_ photocatalyst has been suggested to depend on the injection of electrons from the HOMO of QAD to the CB of TiO_2_ and enhancing the charge separation and consequently boosting of the photocatalytic activity compared to the bare TiO_2_ nanoparticles.

### Supplementary Information

Supplementary Information.

## Data Availability

The datasets used and/or analysed during the current study are available from the corresponding author upon reasonable request.
