# Supplementary material for: Photosensitization of TiO2 microspheres by novel Quinazoline-derivative as visible-light-harvesting antenna for enhanced Rhodamine B photodegradation
Source: Sci Rep. 2023 Aug 9;13:12929. doi: 10.1038/s41598-023-38497-9 (PMC10412568; doi:10.1038/s41598-023-38497-9)
Supplement: Supplementary file 1 — Supplementary Information. [file 41598_2023_38497_MOESM1_ESM.docx]

**Supporting information**

**Photosensitization of TiO_2_ microspheres by novel Quinazoline-derivative as visible-light-harvesting antenna for enhanced Rhodamine B photodegradation^#^**

Mahmoud Adel Hamza^*^, Sameh A. Rizk, Ezz-Elregal M. Ezz-Elregal, Shaimaa A. Abd El-Rahman, Sayed K. Ramadan, Zeinab M. Abou‑Gamra

Chemistry Department, Faculty of Science, Ain-Shams University, Abbassia, Cairo, Egypt

^#^ This article is dedicated as a memorial to **Dr. Alaa Mahmoud Zidan** (our colleague at Ain Shams University) who suddenly passed away on 12 December 2022, she was a role model of a supportive, kind, and modest human before being a promising scientist.

*Corresponding Author E-mail: Mahmoud A. Hamza: [Mahmoud_adel@sci.asu.edu.eg](mailto:Mahmoud_adel@sci.asu.edu.eg)

**List of contents**

| **Figure/Table** | **Page No.** |
| --- | --- |
| **Figure S1.** FTIR of novel Quinazoline-derivative (QAD). | **3** |
| **Figure S2.** ^1^H-NMR of the novel Quinazoline-derivative (QAD) in H_2_O. | **4** |
| **Figure S3.** ^1^H-NMR of the novel Quinazoline-derivative (QAD) in D_2_O. | **5** |
| **Figure S4.** ^13^C-NMR of novel Quinazoline-derivative (QAD). | **6** |
| **Figure S5.** Electron Ionization Mass Spectroscopy (EIMS) of novel Quinazoline-derivative (QAD). | **7** |
| **Figure S6.**  SEM-Mapping spectra and EDX spectrum of the bare TiO_2_ sample | **8** |
| **Figure S7.**  EDX spectrum of the QAD/TiO_2_ sample | **9** |
| **Figure S8.** The room-temperature PL spectra using different excitation wavelengths of the as-synthesized QAD/TiO_2_ compared to the bare TiO_2_ and pure QAD samples. | **10** |
| **Figure S9.** The 1^st^ order plots of the photodegradation of RB dye using the QAD/TiO_2_ nanoparticles under different irradiation sources: UV-A irradiation and Visible Light irradiation. | **11** |
| **Figure S10.** The determination of the point of zero charge (pH_PZC_) using the pH drift method. | **12** |
| **Figure S11.** The temporal UV-Visible spectra of the treated RB photodegraded by QAD/TiO_2_ using different solutions with varying the initial pH values: pH = 2 (a), pH = 4 (b), pH = 6 (c), pH = 8 (d), pH = 10 (e), and pH = 12 (f), under UV-A irradiation | **13** |
| **Figure S12.** The temporal UV-Visible spectra of the treated RB photodegraded using different catalyst loadings of QAD/TiO_2_: 0.25 g/L (a), 0.5 g/L (b), 1 g/L (c), 1.5 g/L (d), 2 g/L (e), and 3 g/L (f), under UV-A irradiation | **14** |
| **Figure S13.** The estimated photodegradation% of RB dye at different irradiation times: 30 min, 60 min and 90 min, achieved by QAD/TiO_2_ using different catalyst loadings (0.25 – 3.00 g/L) under UV-A irradiation | **15** |
| **Figure S14.** The temporal UV-Visible spectra of the treated RB photodegraded by QAD/TiO_2_ using different dye concentration: 0.5 x 10^-5^ M (a), 1 x 10^-5^ M (b), 1.5 x 10^-5^ M (c), and 2 x 10^-5^ M (d), under UV-A irradiation | **16** |
| **Figure S15.** The effect of Oxygen bubbling on the UV-Visible spectra (a,b), (a-x) vs time plots (c), 1^st^ order plots (d) for the photodegradation of RB using QAD/TiO_2_ | **17** |
| **Table S1.** The effect of recyclability of the as-synthesized QAD/TiO_2_ photocatalyst on its photocatalytic activity toward RB photodegradation under UV-A irradiation. | **18** |
| **Table S2.** The effect of scavengers on the RB photodegradation% in 150 min and the corresponding 1^st^ order plots using QAD/TiO_2_ photocatalyst under UV-A irradiation. | **19** |


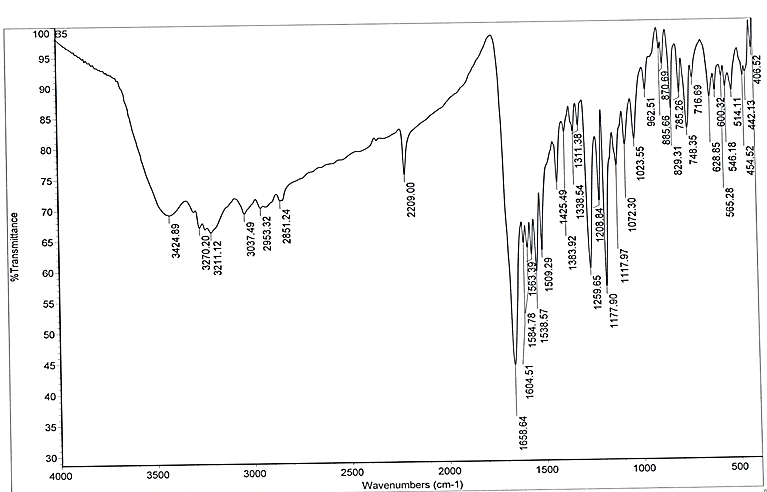


**Figure S1.** FTIR of novel Quinazoline-derivative (QAD).


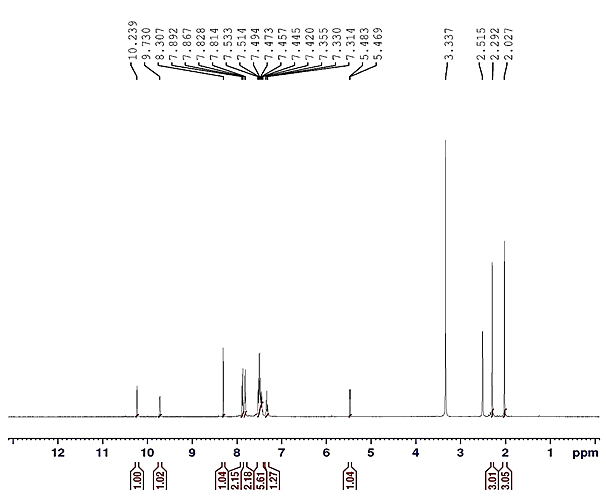


**Figure S2.** ^1^H-NMR of the novel Quinazoline-derivative (QAD) in H_2_O.


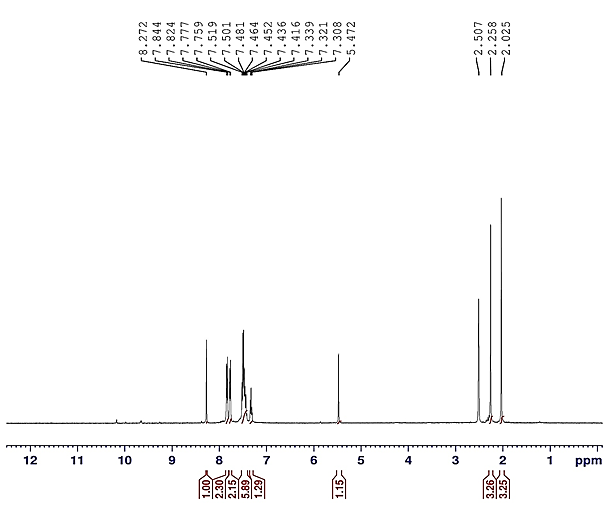


**Figure S3.** ^1^H-NMR of the novel Quinazoline-derivative (QAD) in D_2_O.


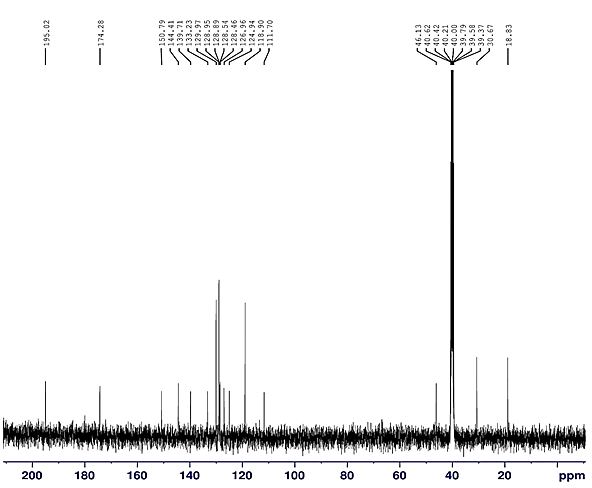


**Figure S4.** ^13^C-NMR of novel Quinazoline-derivative (QAD).


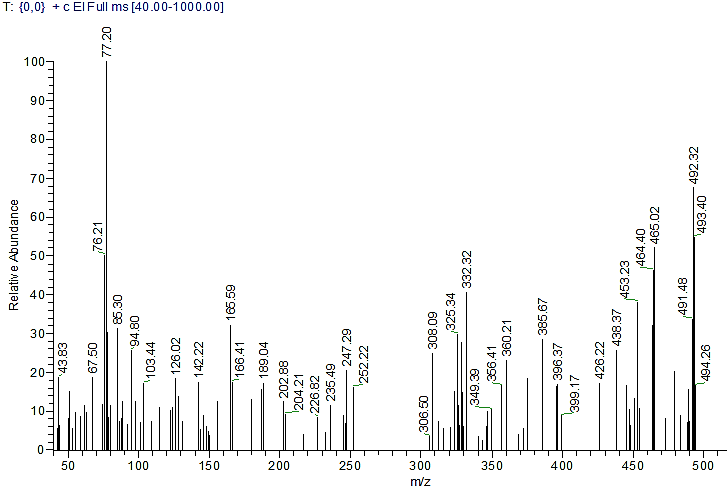


**Figure S5.** Electron Ionization Mass Spectroscopy (EIMS) of novel Quinazoline-derivative (QAD).


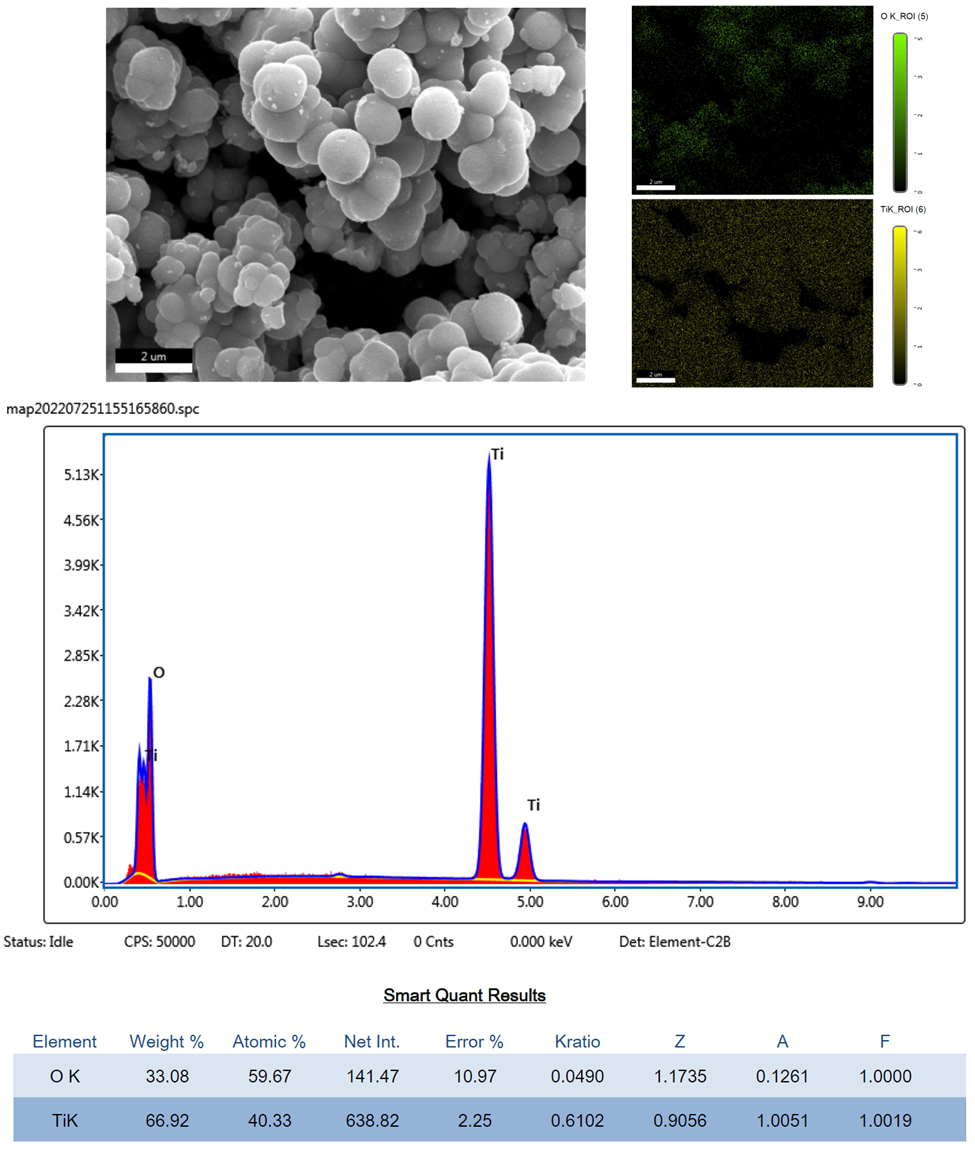


**Figure S6.**  SEM-Mapping spectra and EDX spectrum of the bare TiO_2_ sample


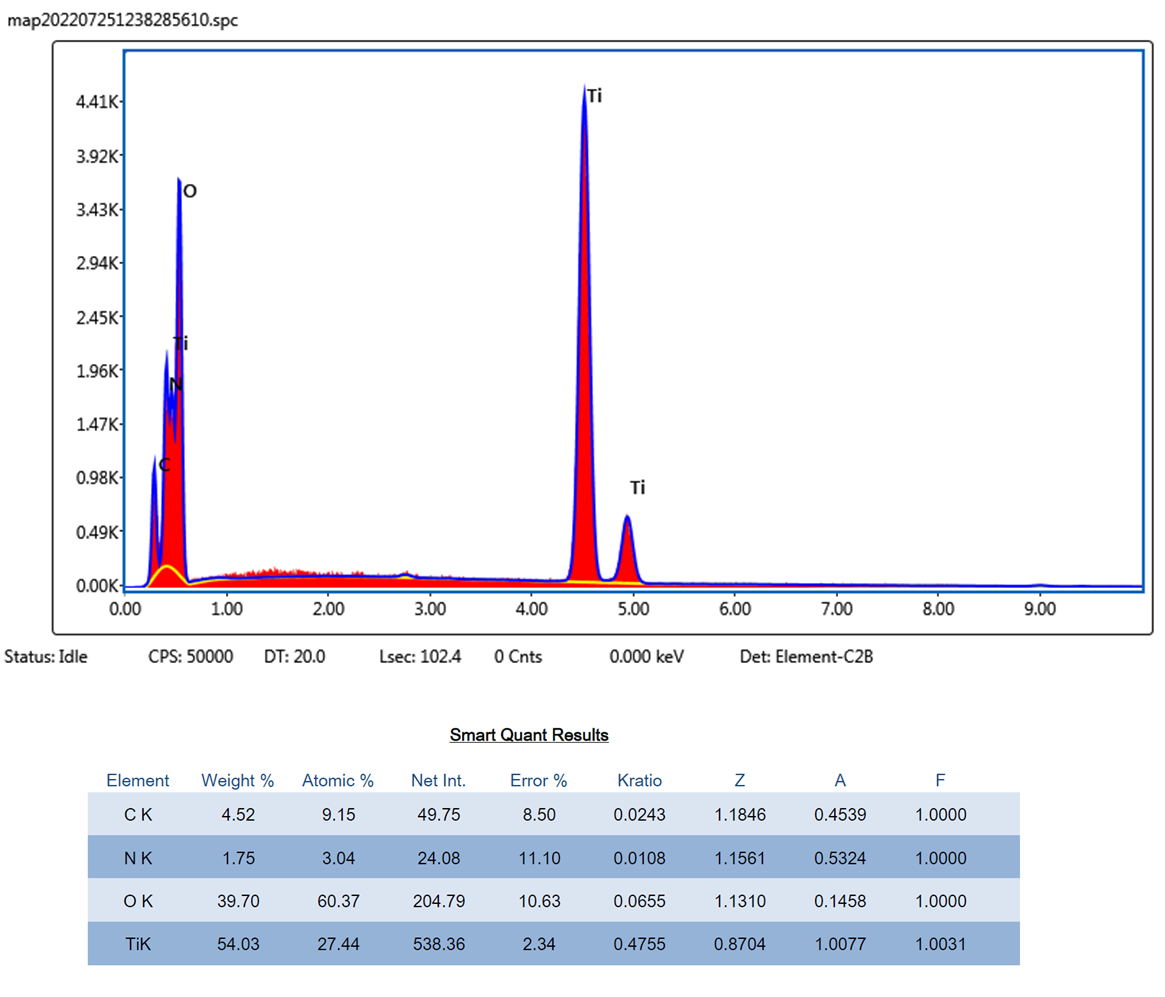


**Figure S7.**  EDX spectrum of the QAD/TiO_2_ sample


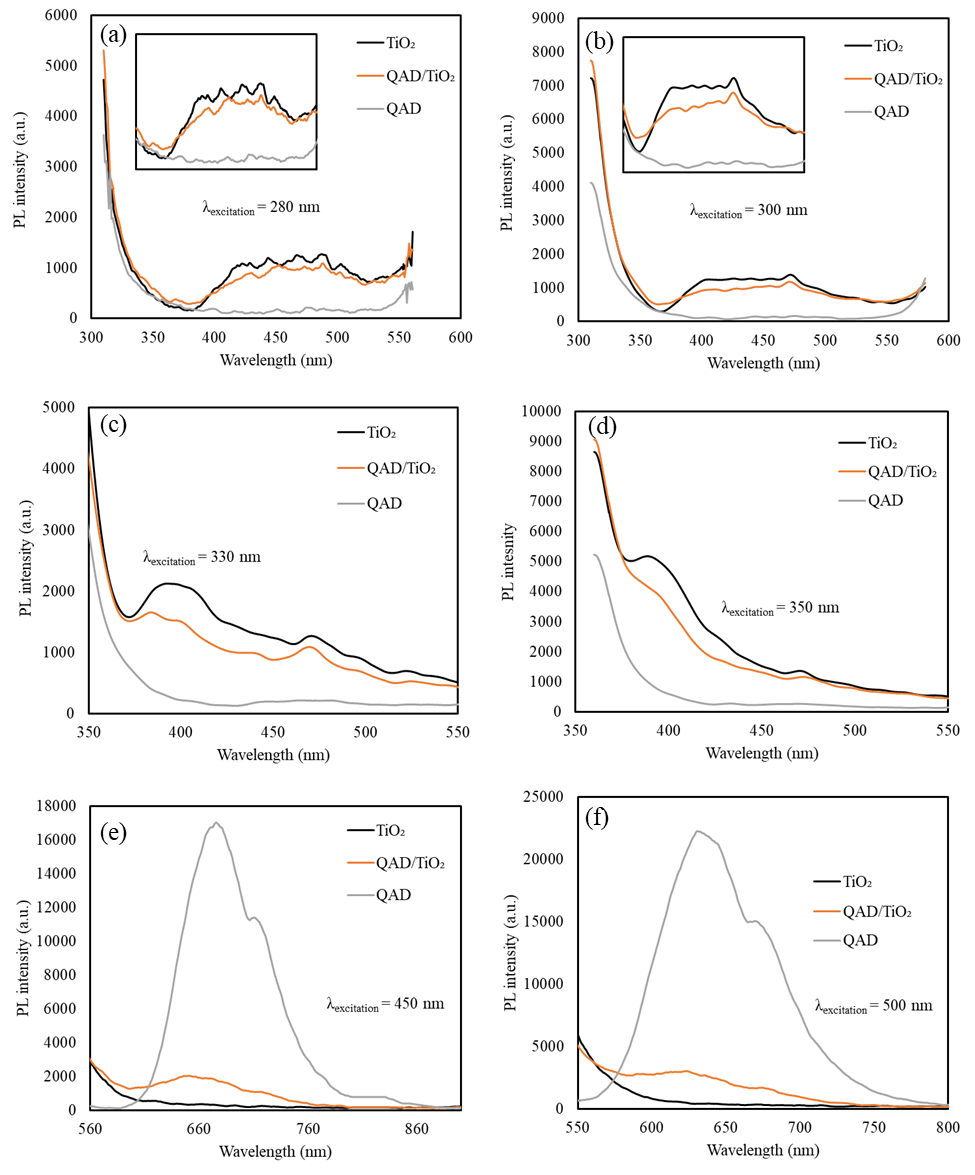


**Figure S8**. The room-temperature PL spectra using different excitation wavelengths of the as-synthesized QAD/TiO_2_ compared to the bare TiO_2_ and pure QAD samples.


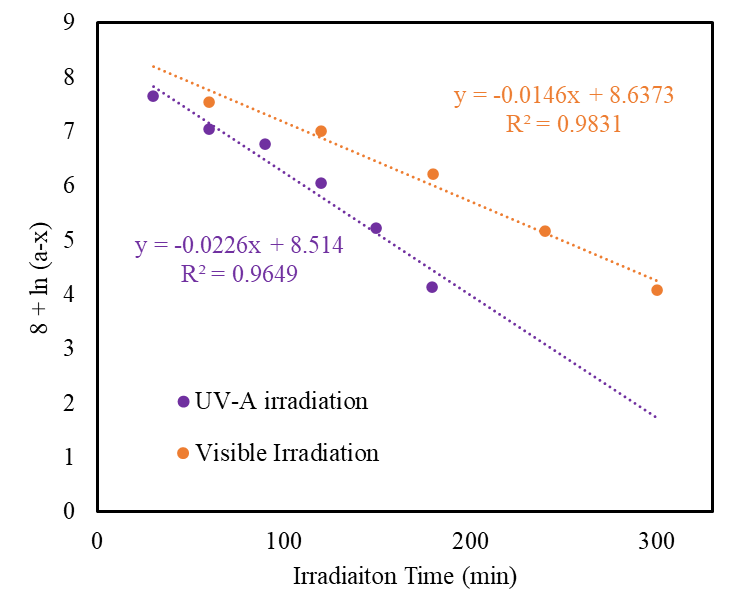


**Figure S9**. The 1^st^ order plots of the photodegradation of RB dye using the QAD/TiO_2_ nanoparticles under different irradiation sources: UV-A irradiation and Visible Light irradiation (Experimental parameters: [Cat]=1 g/L, pH = 6, [RB]_o_ = 1 x 10^-5^ M).


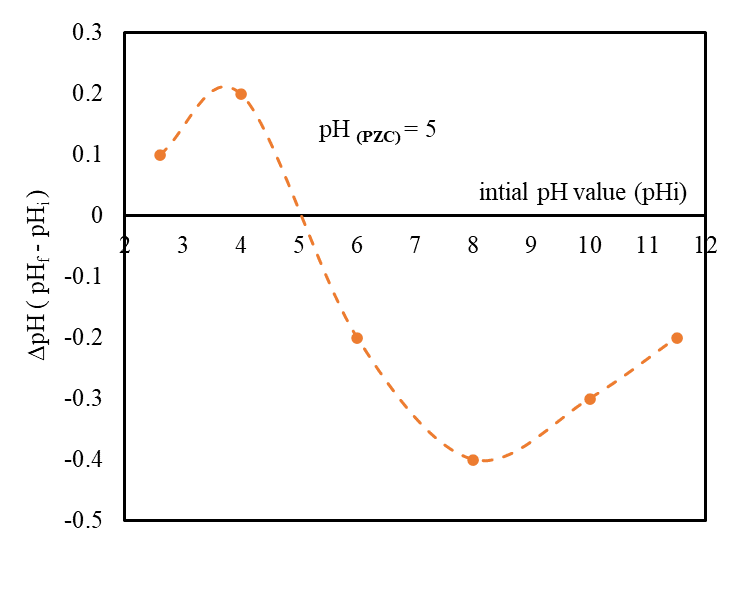


**Figure S10**. The determination of the pH (PZC) using the pH drift method.


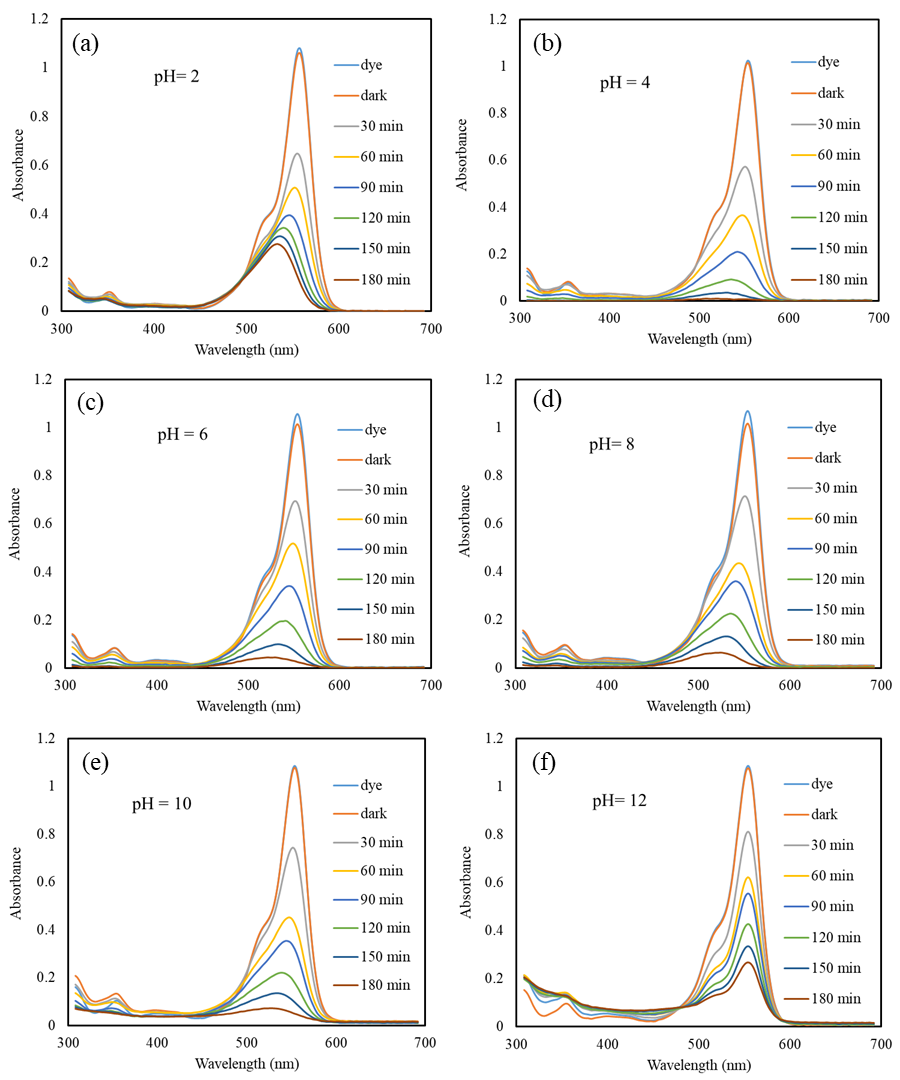
**Figure S11**. The temporal UV-Visible spectra of the treated RB photodegraded by QAD/TiO_2_ using different solutions with varying the initial pH values: pH = 2 (a), pH = 4 (b), pH = 6 (c), pH = 8 (d), pH = 10 (e), and pH = 12 (f), under UV-A irradiation [Other experimental conditions: [Cat] = 1 g/L and [RB]= 1x10^-5^ M]


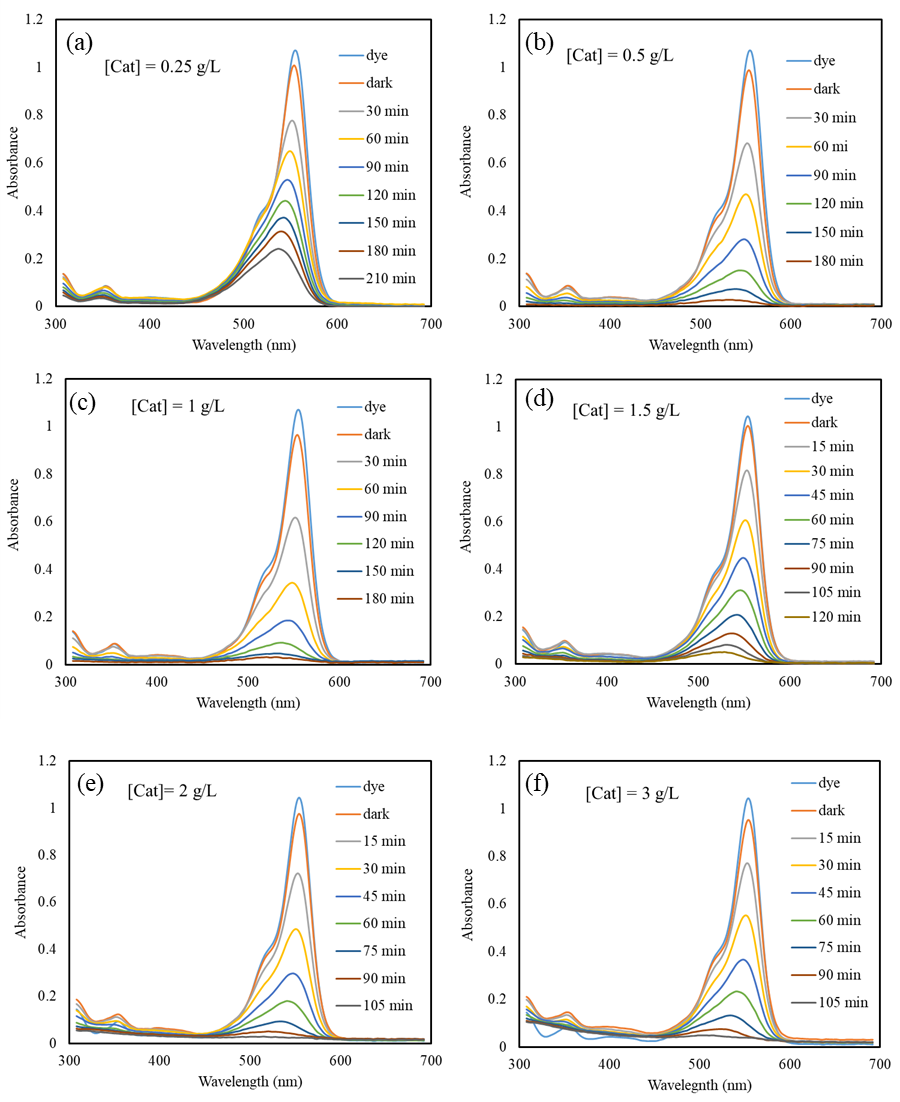


**Figure S12**. The temporal UV-Visible spectra of the treated RB photodegraded using different catalyst loadings of QAD/TiO_2_: 0.25 g/L (a), 0.5 g/L (b), 1 g/L (c), 1.5 g/L (d), 2 g/L (e), and 3 g/L (f), under UV-A irradiation [Other experimental conditions: pH = 4 and [RB]= 1x10^-5^ M]


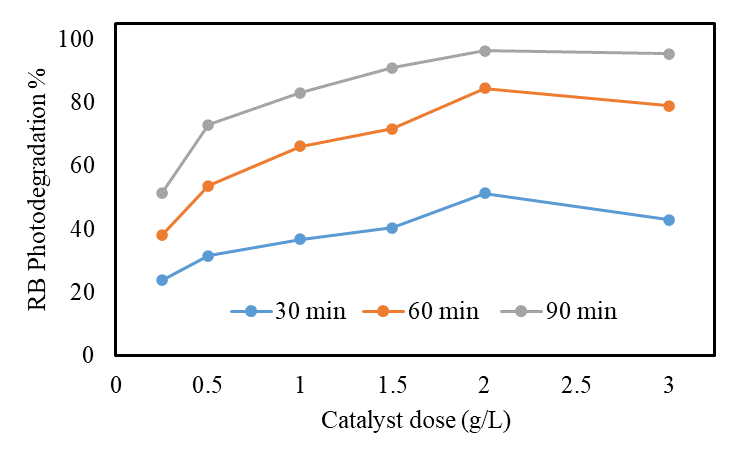


**Figure S13.** The estimated photodegradation% of RB dye at different irradiation times: 30 min, 60 min and 90 min, achieved by QAD/TiO_2_ using different catalyst loadings (0.25 – 3.00 g/L) under UV-A irradiation [Other experimental conditions: pH = 4 and [RB] = 1 x 10^-5^ M]


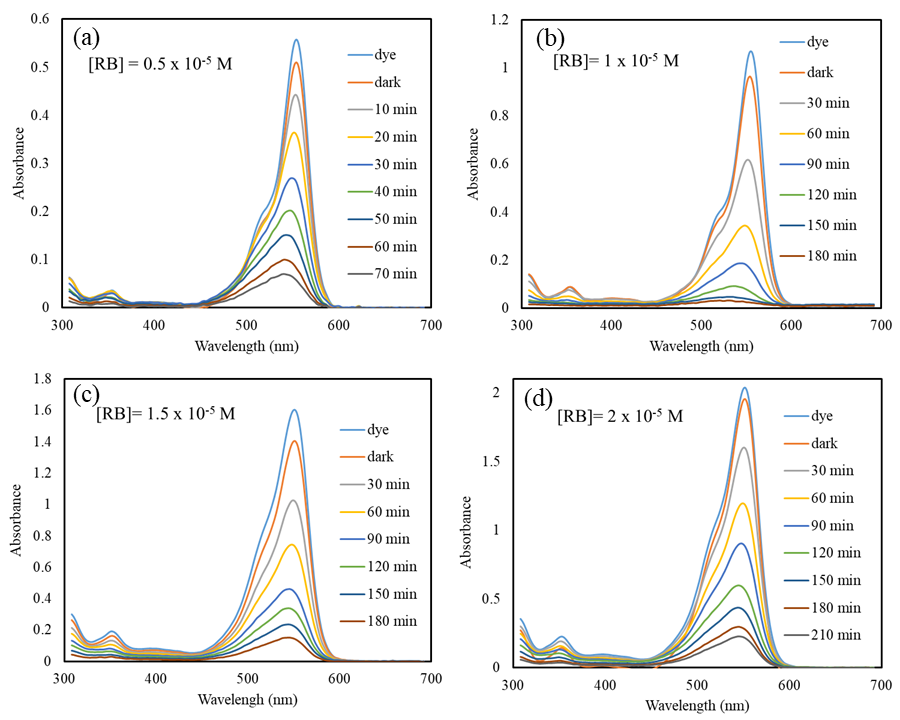


**Figure S14**. The temporal UV-Visible spectra of the treated RB photodegraded by QAD/TiO_2_ using different dye concentration: 0.5 x 10^-5^ M (a), 1 x 10^-5^ M (b), 1.5 x 10^-5^ M (c), and 2 x 10^-5^ M (d), under UV-A irradiation [Other experimental conditions: pH = 4 and [Cat]= 1 g/L]


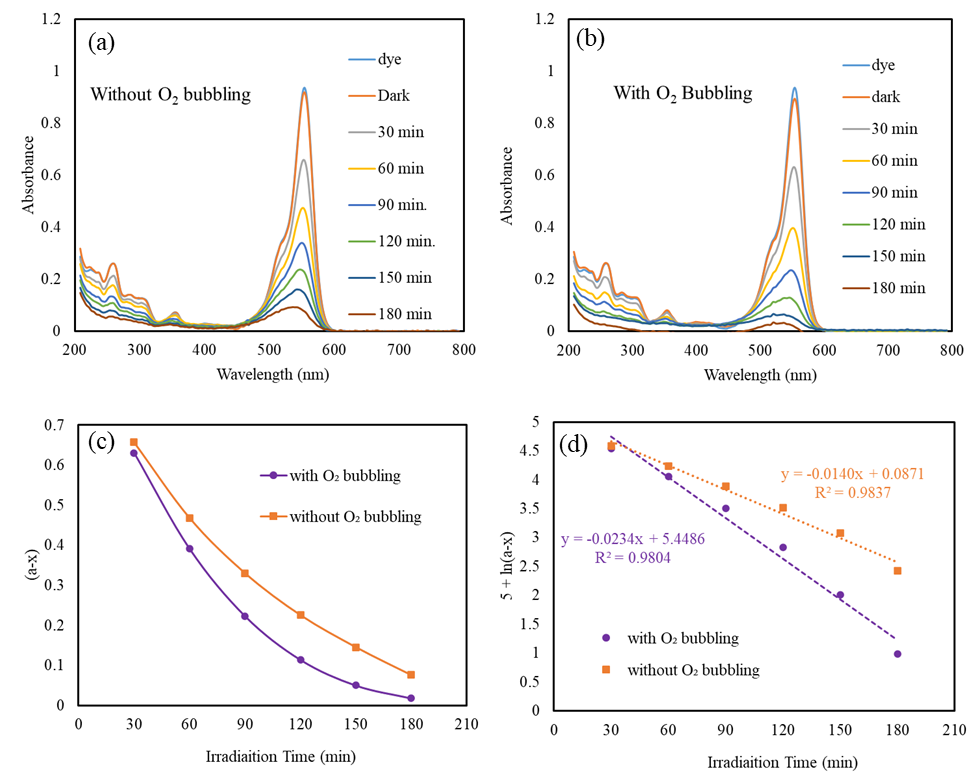


**Figure S15**. The effect of Oxygen bubbling on the UV-Visible spectra (a,b), (a-x) vs time plots (c), 1^st^ order plots (d) for the photodegradation of RB using QAD/TiO_2_ [experimental conditions: pH = 4 and [Cat]= 1 g/L, [RB] = 1x10^-5^ M]

**Table S1.** The effect of recyclability of the as-synthesized QAD/TiO_2_ photocatalyst on its photocatalytic activity toward RB photodegradation under UV-A irradiation (Experimental parameters: [Cat] = 1 g/L, pH = 4, [RB] = 1 x 10^-5^ M, dark time = 1 h, UV-irradiation time = 3 h for each cycle).

|  | **RB removal %** | | | | |
| --- | --- | --- | --- | --- | --- |
| Cycle number | **Exp1** | **Exp 2** | **Average** | **Standard deviation** | ***Standard error*** |
| 1 | 93.54 | 96.09 | 94.82 | 1.80 | 1.27 |
| 2 | 88.57 | 90.88 | 89.72 | 1.63 | 1.16 |
| 3 | 85.08 | 89.02 | 87.05 | 2.79 | 1.97 |
| 4 | 79.44 | 82.6 | 81.02 | 2.23 | 1.58 |

**Table S2.** The effect of scavengers on the RB photodegradation% in 150 min and the corresponding 1^st^ order rate constants using QAD/TiO_2_ photocatalyst under UV-A irradiation (Experimental parameters: [Cat] = 1 g/L, pH = 4, [RB] = 1 x 10^-5^ M, [Scavenger] = 1x10^-5^ M)

| **Scavenger** | **RB removal%** | | | **Standard deviation** | **Standard error** |
| --- | --- | --- | --- | --- | --- |
|  | **Exp. 1** | **Exp. 2** | **Average** |  |  |
| Without Scavenger | 98.17 | 98.63 | 96.58 | 2.24 | 1.58 |
| MeI | 83.77 | 80.25 | 82.01 | 3.52 | 2.49 |
| i-PrOH | 94.77 | 94.04 | 94.40 | 0.73 | 0.52 |
| MV | 83.77 | 80.25 | 82.01 | 3.52 | 2.49 |
| p-BQ | 57.32 | 58.14 | 57.73 | 0.81 | 0.58 |
| **Scavenger** | ***k_obs_* (min^-1^)** | | | **Standard deviation** | **Standard error** |
|  | **Exp. 1** | **Exp. 2** | **Average** |  |  |
| Without Scavenger | 0.0249 | 0.0179 | 0.0214 | 0.004949 | 0.00350 |
| MeI | 0.0149 | 0.0134 | 0.01415 | 0.001061 | 0.00075 |
| i-PrOH | 0.0210 | 0.0203 | 0.02065 | 0.000495 | 0.00035 |
| MV | 0.0125 | 0.0125 | 0.0125 | 0 | 0 |
| p-BQ | 0.0052 | 0.0052 | 0.0052 | 0 | 0 |
